# Novel microRNAs modulating ecto-5′-nucleotidase expression

**DOI:** 10.3389/fimmu.2023.1199374

**Published:** 2023-06-20

**Authors:** Theresa Kordaß, Tsu-Yang Chao, Wolfram Osen, Stefan B. Eichmüller

**Affiliations:** ^1^ GMP & T Cell Therapy Unit, German Cancer Research Center (DKFZ), Heidelberg, Germany; ^2^ Faculty of Biosciences, University Heidelberg, Heidelberg, Germany

**Keywords:** miRNAs, NT5E/CD73, CD274, ENTPD1, melanoma, breast cancer

## Abstract

**Introduction:**

The expression of immune checkpoint molecules (ICMs) by cancer cells is known to counteract tumor-reactive immune responses, thereby promoting tumor immune escape. For example, upregulated expression of ecto-5′-nucleotidase (NT5E), also designated as CD73, increases extracellular levels of immunosuppressive adenosine, which inhibits tumor attack by activated T cells. MicroRNAs (miRNAs) are small non-coding RNAs that regulate gene expression at the post-transcriptional level. Thus, the binding of miRNAs to the 3′-untranslated region of target mRNAs either blocks translation or induces degradation of the targeted mRNA. Cancer cells often exhibit aberrant miRNA expression profiles; hence, tumor-derived miRNAs have been used as biomarkers for early tumor detection.

**Methods:**

In this study, we screened a human miRNA library and identified miRNAs affecting the expression of ICMs NT5E, ENTPD1, and CD274 in the human tumor cell lines SK-Mel-28 (melanoma) and MDA-MB-231 (breast cancer). Thereby, a set of potential tumor-suppressor miRNAs that decreased ICM expression in these cell lines was defined. Notably, this study also introduces a group of potential oncogenic miRNAs that cause increased ICM expression and presents the possible underlying mechanisms. The results of high-throughput screening of miRNAs affecting NT5E expression were validated *in vitro* in 12 cell lines of various tumor entities.

**Results:**

As result, miR-1285-5p, miR-155-5p, and miR-3134 were found to be the most potent inhibitors of NT5E expression, while miR-134-3p, miR-6859-3p, miR-6514-3p, and miR-224-3p were identified as miRNAs that strongly enhanced NT5E expression levels.

**Discussion:**

The miRNAs identified might have clinical relevance as potential therapeutic agents and biomarkers or therapeutic targets, respectively.

## 1 Introduction

The significance of microRNAs (miRNAs) in cancer development and progression has become increasingly evident since the first miRNA molecule was described by Lee et al. three decades ago (1). miRNAs are highly conserved small non-coding RNA molecules, 19 to 25 nucleotides in length (2) that regulate cellular processes such as proliferation, differentiation, metabolism, and apoptosis (3, 4). According to miRbase, approximately 2,000 mature miRNA sequences have been identified in mice, whereas 2,654 miRNAs have been identified in humans (miRbase database release 22.1, October 2018) (5). Together with Ago proteins, the mature miRNA sequence forms a miRNA-induced silencing complex, which binds to respective mRNA species, thereby affecting the stability and translation rate of the targeted mRNA molecule (6). Most miRNAs bind with their seed sequence, comprising 5–6 nucleotides, to the 3′-untranslated region (3′-UTR) of the mRNA molecule (7, 8). Due to the small size of the seed sequence, each miRNA can target multiple mRNAs, thereby regulating a variety of cellular pathways and networks. Conversely, a given mRNA molecule can be targeted by various miRNAs. Hence, miRNA-mediated regulation of gene expression is an extremely complex mechanism that simultaneously affects multiple targets and regulators (9, 10). In addition to the inhibition of translation or degradation of the targeted mRNA as a consequence of the 3′-UTR association, miRNAs may also bind to the 5′-UTR of target mRNAs or interact with the coding DNA sequence of target genes (11, 12). miRNAs are also capable of enhancing target gene expression via direct interaction with promoters or enhancer regions (13, 14).

miRNA expression profile analysis performed on human tumor types has provided a useful tool to unravel the functional bias of degenerated regulation of gene and protein expression in cancer cells. Furthermore, differential miRNA expression levels in healthy tissues *vs* tumors demonstrated the power of miRNA signatures as biomarkers for cancer diagnosis and staging. In fact, a signature based on the plasma levels of 38 miRNAs, called the MEL38 signature, has been used to classify high-risk and low-risk melanomas (15).

Tumor cells are characterized by dysregulated miRNA expression patterns. Thus, miRNAs with tumor-suppressive or tumor-promoting capacities have been identified in cancer cells and are designated as tumor suppressor miRNAs (tsmiRs) or oncogenic miRNAs (oncomiRs), respectively (16). tsmiRs are generally underrepresented in cancer cells, and the loss of tsmiR expression in healthy cells can be a starting point for cancer onset and progression. For example, low levels of miR-193a-3/5p were found in the tumor tissues and exosomes of cutaneous melanoma patients. However, re-expression of miR-193a-3/5p reduced the viability of various melanoma cell lines by targeting *KRAS*, *MTOR*, and *MCL1*, thus highlighting the tumor-suppressive role of this miRNA (17). Conversely, the expression levels of oncomiRs are often elevated in cancer cells. For instance, miR-519a expression is strongly enhanced in tamoxifen-resistant ER^+^ breast cancer cells. Furthermore, miR-519a was found to promote the proliferation of cancer cells through anti-apoptotic functions mediated by direct targeting of the tumor suppressor genes *PTEN*, *RB1*, and *CDKN1A* (18).

Cancer cells are known to evade the immune system by expressing inhibitory immune checkpoint molecules (ICMs), whose primordial function consists of the maintenance of self-tolerance and protection from overshooting immune responses (19). Notably, miRNAs can affect processes of immune escape in cancer cells in either direction. On the one hand, oncomiRs causing dysregulated expression of ICMs or molecules belonging to the antigen-presentation machinery (20) have been described. Conversely, tsmiRs, such as miR-497 and miR-195, were found to inhibit the expression of the immune checkpoint molecule *CD274* (21) through direct 3′-UTR interactions, thereby sustaining tumor reactive T cell responses.

Additionally, due to its tumor-promoting effects on proliferation, miR-519a-3p can also be considered an oncomiR involved in immune modulatory processes. Advanced stage breast cancer with mutated p53 has been shown to exhibit high expression of miR-519a-3p, which is correlated with unfavorable clinical outcomes. Breunig et al. showed that miR-519a-3p expression diminished killing of breast cancer cells by natural killer (NK) cells by inhibiting the ligands for the NK cell-activating receptor NKG2D. miR-519a-3p expression allows breast cancer cells to evade NK cell-mediated killing by lowering the cell surface expression of NKG2D ligands MICA and ULBP2, which are direct targets of this oncomiR (22).

One important checkpoint molecule involved in immune escape is the ectonucleotidase CD73/NT5E. Upregulated CD73/NT5E expression in cancer cells results in the accumulation of immunosuppressive adenosine, which dampens the function of adenosine receptor (A2A)-expressing T cells and NK cells present in the tumor microenvironment (23). In fact, monoclonal antibodies or small-molecule inhibitors that block CD73 function have been considered for immune checkpoint therapy (24–29) and are currently being investigated for the treatment of metastatic breast cancer, gastrointestinal cancer, and other advanced solid tumors in clinical trials (29). Therefore, the current study identified and characterized novel miRNAs that affect the expression of various ICMs, with a particular focus on the immune checkpoint molecule NT5E/CD73. Established ICM-suppressive miRNAs, including species inhibiting NT5E, might open new options for cancer therapy, while the knowledge of miRNAs driving NT5E expression should provide deeper insights into the mechanisms of cancer progression and immune escape.

## 2 Materials and methods

### 2.1 Cell lines and cell culture

All cell lines used in this study were cultured in RPMI medium supplemented with 10% FBS without antibiotics at 37°C and 5% CO_2_. The supernatants of the cultured cells were regularly checked for mycoplasma contamination by PCR. DNA fingerprinting was performed to confirm the authenticity of human tumor cell lines by the Forensic Medicine Department of the Heidelberg University Hospital (Heidelberg, Germany). The purchased cell lines were fingerprinted upon receipt. The breast cancer cell line MDA-MB-231, colon cancer cell line HCT-116, and melanoma cell line SK-Mel-28 were purchased from ATCC. The melanoma cell lines used in this study were MaMel-02/-05/-26a/-42/-53a/-57/-61a/-68 and -73a. These cell lines were authenticated by STR profiling as described elsewhere (30).

### 2.2 miRNA library screen

The human microRNA Mimic Version 21 library containing 2,754 miRNAs allotted in 36 96-well plates was used for screening. Each plate contained two negative control miRNAs: ath-miR-416 (control 1) and cel-miR-243 (control 2). To prepare library plates for transfection, the initial library plates were centrifuged and miRNAs were dissolved in RNAse-free water to generate a 20 µM stock solution. The stock solution was subsequently diluted in a new 96-well plate to give a 2 µM working solution, which was used for transfection. Transfection of the breast cancer cell line MDA-MB-231 and melanoma cell line SK-Mel-28 was performed with RNAi Lipofectamine Max reagent according to the manufacturer’s protocol in a 96-well format. Therefore, 2.5 × 10^4^ cells were seeded in flat-bottom 96-well culture plates and cultured for 24 h. Subsequently, the cells were transfected with a final miRNA concentration of 25 nM per well. Fresh medium (100 µL) was added to each well at 24 h post-transfection. The medium was replaced with 200 µL of fresh medium 48 h post-transfection. At 72 h post-transfection, the cells were harvested and stained with fluorochrome-conjugated antibodies for flow cytometric analysis. The Live/Dead Fixable Yellow Dead Cell Stain Kit (Life Technologies, Carlsbad, CA, USA) was used to exclude dead cells, and a phycoerythrin-conjugated antibody was used to detect NT5E surface expression. ENTPD1 surface expression in SK-Mel-28 cells was measured with BB515 conjugated antibody and CD274 expression in MDA-MB-231 cells was analyzed with a phycoerythrin-Cy7 conjugated antibody. All antibodies used are listed in **Supplementary Table S1**. Pacific orange was used at 1:1000 dilution. ENTPD1-and CD274-specific fluorochrome-conjugated antibodies were used at a 1:100 dilution. The NT5E-specific antibody conjugate was diluted to 1:400 for use. Staining mixture (100 µL) was supplied to each well and cells were stained for 1 h at 4°C protected from light. After three washing steps cells were resuspended in 100 µL FACS buffer (PBS + 3% FCS). Cells were passed through a mesh of MultiScreen-MESH Filter 96-well plates to remove doublets. FACS measurements of 96-well plates were run on BD LSRFortessa™ Flow Cytometer (Becton Dickinson, Franklin Lakes, USA) equipped with a high-throughput screening system. FlowJo version 10 was used to analyze the acquired data. Acquisition gates were set on single live cells. The gates for the fluorochrome channels were set according to respective isotype controls. Transfection with siRNAs targeting NT5E or ENTPD1 was performed in each plate to monitor transfection efficacy. For each plate, the median fluorescence intensity (MFI) values of the miRNA transfectants were exported to FlowJo. Subsequent data analyses were conducted using R and RStudio Version 3.5.1. To allow for the comparison of individual screening plates, the MFI values were z-score normalized for each plate and each channel with the scale function from the R package. Untreated cells, cells incubated with isotype controls, and empty wells were excluded prior to normalization. The Z-score normalization sets the mean value of each plate to zero and standard deviation to one. To identify miRNAs with the strongest effect on NT5E, ENTPD1, or CD274 expression, z-scores from all plates were ranked. Effects with a z-score ≥ │1.645│ were considered as significant.

### 2.3 Malachite green assay

To assess the effects of the selected miRNAs on the enzymatic activity of NT5E, a modified protocol based on the method published by Allard et al. (31) was applied. Briefly, the colorimetric malachite green assay was used to quantify the inorganic phosphate released through the hydrolysis of AMP to adenosine. The inorganic phosphate reacted with malachite green molybdate under acidic conditions and was quantified by colorimetric determination of absorbance at 620 nm. The amount of phosphate released is directly correlated with the enzymatic activity of NT5E. We seeded 2 × 10^5^ cells per well in 12 well plates followed by incubation at 37°C/5% CO_2_ for 24 h, until transfection with 50 nM miRNA/siRNA. After 48 or 72 h, the cells were washed extensively with phosphate-free buffer (**Supplementary Table S3**). Then, 500 µL phosphate-free buffer was added to each well, and cells were incubated for 15 min at 37°C. A 50 mM AMP working solution was freshly prepared from 1 M frozen stock solution, and cells were supplemented with a final concentration of 400 µM AMP per well. After 30 min of incubation at 37°C, 25 µL of the supernatant was transferred to a 96-well read out plate, filled with 25 µL 10 mM EDTA per well. Quintuplicates were performed for each condition. The Malachite Green PO4 Detection Kit (R&D Systems, Minneapolis, MN, USA) was used according to the manufacturer’s protocol. Briefly, 10 µL of Malachite Green Reagent A was added to each well, and the plates were incubated on a microplate shaker for 10 min. Subsequently, 10 µL Malachite Green Reagent B was added to each well. Following incubation for 5 min, absorbance was measured at 620 nm using a CLARIOstar Plus spectrophotometer (BMG Labtech, Ortenberg, Germany).

### 2.4 Luciferase-reporter assays

The pLS-NT5E-3′-UTR plasmid was purchased from Active Motif (La Hulpe, Belgium). This plasmid contains the NT5E 3′-UTR fused to the luciferase reporter gene and enables direct binding of miRNAs to the NT5E 3′-UTR. Therefore, 1 × 10^4^ cells were seeded per well in flat-bottom 96-well plates and co-transfected after 24 h with 25 nM miRNA and 100 ng plasmid (pLS-NT5E-3′UTR or mutated versions) using the DharmaFect Duo reagent (GE Dharmacon, Lafayette, USA). Luciferase signal intensity was measured 24 h pos -transfection using the LightSwitch™ Luciferase Assay Reagent (Active Motif, La Hulpe, Belgium) according to the manufacturer’s protocol. Luminescence values were normalized to the respective mimic control-1 transfected samples. Quintuplicates were performed for each condition. To prove direct miRNA binding to the NT5E 3′-UTR, the respective binding sites within the NT5E 3′-UTR were mutated using a Quick Change II site-directed mutagenesis kit, according to the manufacturer’s protocol (Agilent, Santa Clara, USA). The QuickChange Primer Design webtool was used to design primers for the deletion of single nucleotides within the NT5E 3′-UTR (https://www.agilent.com/store/primerDesignProgram.jsp). All primers used are listed in **Supplementary Table S10**.

### 2.5 Microarray analysis

Gene expression profiling of tumor cell lines transfected with miRNAs enhancing NT5E expression was performed as follows: SK-Mel-28, MDA-MB-231, and MaMel-02 cells were transfected with 50 nM miRNA, including a non-targeting control miRNA (control-1/ath-miR-416) in a 12 well plate format, performing triplicates for each format. After 48 h, cells were harvested for RNA isolation using an RNAeasy kit (Qiagen, Hilden, Germany). Microarrays were performed by DKFZ-Genomics and Proteomics Core Facility using an Affymetrix Clariom S human chip for all samples. Raw data processing was also performed by the Core Facility. Differential gene expression was calculated by comparing miRNA-induced gene expression levels to the respective gene expression levels following mimic control-1 transfection. Raw and normalized data were uploaded to Gene Expression Omnibus GSE228642; https://www.ncbi.nlm.nih.gov/geo/query/acc.cgi?acc=GSE228642). Venn diagrams were generated using a web tool from the Bioinformatics and Evolutionary Genomics Department of Ghent University (http://bioinformatics.psb.ugent.be/webtools/Venn/).

## 3 Results

### 3.1 Prediction of miRNAs targeting multiple ICMs

To investigate the miRNAs involved in the regulation of ICM expression, we first performed a combined *in silico* analysis utilizing ten databases predicting miRNA-target interactions relevant for the expression of CD274, CTLA4, ENTPD1, and NT5E (**Supplementary Methods**). As the prediction tools applied were based on different computational concepts, only the predicted miRNA/mRNA target interactions shared by at least three databases were considered. Using this approach, we identified 44 miRNAs with a strong evidence of NT5E targeting (**Supplementary Table S4**). Furthermore, 39 miRNAs were predicted to target CD274 (**Supplementary Table S5**), while 33 miRNAs were identified as putative CTLA4 targeting candidates (**Supplementary Table S6**). Only three candidate miRNAs were predicted to target ENTPD1 (**Supplementary Table S7**). Therefore, less stringent selection criteria were applied, and candidate miRNAs predicted using at least two resources were selected for further investigation. Focusing on miRNAs predicted to target all four ICMs (**Supplementary Table S8**), we identified 49 miRNAs with a potential 3′-UTR binding site shared by mRNA molecules encoding CD274, CTLA4, ENTPD1, and NT5E, for example, miR-422a, miR-155, and miR-193a/b.

### 3.2 Comprehensive miRNA library screen reveals miRNAs modulating ICM expression in human cancer cell lines

Based on the gene expression levels reported in the NCI-60 dataset, the melanoma cell line SK-Mel-28 and breast cancer cell line MDA-MB-231 were selected as NT5E expressing human tumor cell lines for the library screen (**Supplementary Figure S1**). According to the NCI-60 database, no further ICMs involved in CTL recognition were expressed by these cell lines. However, SK-Mel-28 cells co-expressed ENTPD1 in addition to NT5E (**Supplementary Figure S2**). Notably, both enzymes work together, producing immunosuppressive adenosine upon hydrolysis of ATP in a multi-step reaction (32). Moreover, MDA-MB-231 cells showed surface expression of the checkpoint molecule CD274, in addition to NT5E (**Supplementary Figure S2**). Thus, during the miRNA library screening, surface expression levels of both NT5E and ENTPD1 were measured in SK-Mel-28 cells, while co-expression of CD274 and NT5E was monitored in MDA-MB-231 cells, and 2,754 miRNAs were screened for their effects on the surface expression of the selected ICMs (**Supplementary Figures S1**, **S3**).

**Figure 1:**
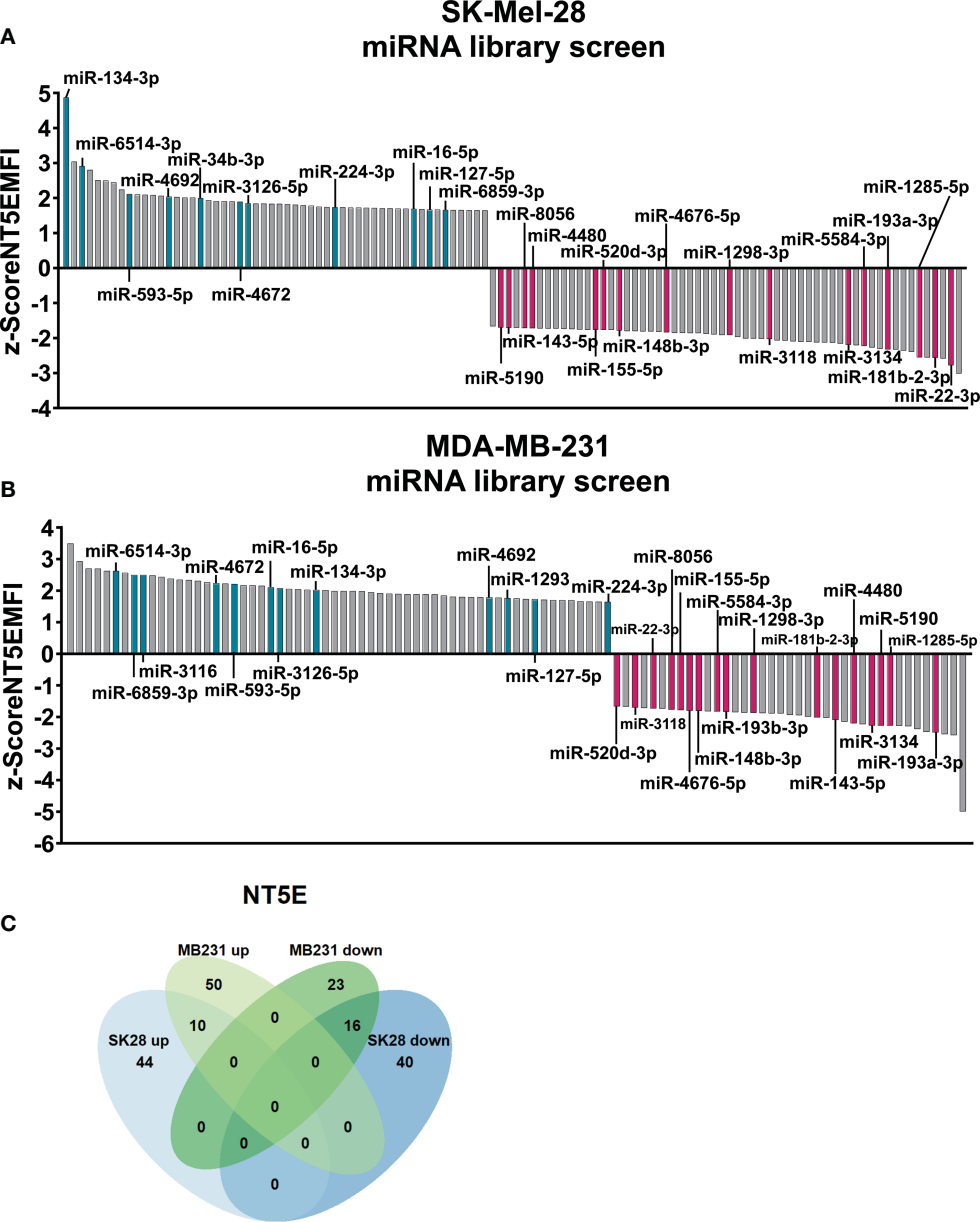
Comprehensive microRNA (miRNA) library screen reveals miRNAs affecting ecto-5′-nucleotidase (NT5E) surface expression in human tumor cell lines. The human melanoma cell line SK-Mel-28 **(A)** and human breast cancer cell line MDA-MB-231 **(B)** were transfected with a human miRNA library and changes in NT5E surface expression was measured by flow cytometry 72 h post transfection. The median fluorescence intensity values (MFI) were z-score normalized for each plate. Z-Scores ≥│1.645│ were considered as significant changes. Modulating miRNAs selected for further validation are depicted in turquois (enhancing NT5E expression) and magenta (decreasing NT5E expression). **(C)** Venn diagram of miRNAs with significant effects on NT5E surface expression in MDA-MB-231 (MB231) and SK-Mel-28 (SK28) cells used for the screen. miRNAs showing significant effects in both cell lines are depicted.

Regarding SK-Mel-28 cells, our screening results revealed 54 miRNAs that significantly enhanced NT5E surface expression, whereas 56 miRNAs led to reduced NT5E expression levels (**Figure 1A**). The miRNAs showing modulating effects beyond the threshold (z-score > |2|) are listed in **Table 1**. Notably, 18 miRNAs were identified that strongly increased NT5E surface expression. Moreover, the NT5E 3′-UTR harbored potential miRNA binding sites for seven of these activating miRNAs. In contrast, our screening revealed 27 miRNAs that strongly decreased NT5E surface levels. In line with this, the NT5E 3′-UTR contained at least one potential binding site for 20 of these miRNAs. We noted that NT5E inhibiting miRNAs turned out more often as potential binders of the NT5E 3′-UTR than NT5E activating miRNAs (p = 0.0296, two-sided Fisher’s exact test).

**Table 1:** miRNAs affecting NT5E surface expression of SK-Mel-28 cells.

miRNA	z-score	BS	miRNA	z-score	BS
**miR-134-3p**	4.87	0	**miR-6787-3p**	-3.01	0
**miR-7854-3p**	3.04	1	**miR-22-3p**	-2.78	1
**miR-6514-3p**	2.91	0	**miR-203a-5p**	-2.58	2
**miR-6132**	2.80	0	**miR-181b-2-3p**	-2.56	0
**miR-146b-3p**	2.51	0	**miR-92b-3p**	-2.56	1
**miR-3152-5p**	2.50	0	**miR-1285-5p**	-2.55	2
**miR-6736-3p**	2.45	0	**miR-6795-3p**	-2.39	0
**miR-3605-5p**	2.24	1	**miR-6888-3p**	-2.36	2
**miR-593-5p**	2.11	0	**miR-921**	-2.33	0
**miR-5697**	2.10	1	**miR-193a-3p**	-2.32	1
**miR-548au-3p**	2.09	0	**miR-4647**	-2.30	1
**miR-34b-5p**	2.08	3	**miR-6876-3p**	-2.27	3
**miR-6818-5p**	2.06	1	**miR-5584-3p**	-2.22	2
**miR-4692**	2.04	1	**miR-4714-5p**	-2.20	1
**miR-555**	2.03	0	**miR-3134**	-2.19	2
**miR-516b-5p**	2.01	1	**miR-6780a-3p**	-2.15	0
**miR-520a-5p**	2.01	0	**miR-6759-3p**	-2.13	0
**miR-34b-3p**	2.00	0	**miR-373-3p**	-2.12	1
			**miR-3176**	-2.10	2
			**miR-3942-3p**	-2.10	1
			**miR-6820-3p**	-2.09	4
			**miR-548t-3p**	-2.07	1
			**miR-199a-5p**	-2.07	0
			**miR-3118**	-2.03	2
			**miR-578**	-2.02	1
			**miR-885-5p**	-2.01	1
			**miR-124-3p**	-2.01	3

Only miRNAs with a z-score > |2.0| are listed. The number of potential binding sites within the NT5E 3′-UTR were obtained by miRmap.

Analysis of miRNA-transfected MDA-MB-231 cells revealed that 60 miRNAs increased NT5E surface expression, while 39 miRNAs significantly diminished NT5E expression levels (**Figure 1B**). miRNAs mediating changes above the threshold in NT5E surface expression in MDA-MB-231 cells (z-score > |2|) are listed in **Table 2**. Among the 29 miRNAs that increased NT5E surface expression beyond a z-score of 2, five miRNA candidates were predicted to encounter at least one binding site within the NT5E 3′-UTR. Of the 17 miRNAs that strongly reduced NT5E expression, 11 were predicted to bind to the NT5E 3′-UTR. Similarly, as observed for SK-Mel-28 cells, the NT5E inhibiting miRNAs represented potential binders of the NT5E 3′-UTR more often compared to NT5E activating miRNAs (p = 0.0030, two-sided Fisher’s exact test) in the case of the MDA-MB-231 cell line.

**Table 2:** miRNAs affecting NT5E surface levels of MDA-MB-231 cells.

miRNA	z-score	BS	miRNA	z-score	BS
**miR-196a-3p**	3.49	0	**miR-512-3p**	-4.99	1
**miR-5589-3p**	2.93	1	**miR-2467-3p**	-2.57	4
**miR-181d-3p**	2.70	0	**miR-455-5p**	-2.54	0
**miR-3189-3p**	2.70	0	**miR-193a-3p**	-2.48	1
**miR-4673**	2.63	0	**miR-376c-5p**	-2.47	1
**miR-6514-3p**	2.63	0	**miR-659-3p**	-2.38	0
**miR-191-5p**	2.57	0	**miR-203b-3p**	-2.29	0
**miR-6859-3p**	2.50	0	**miR-376b-5p**	-2.28	1
**miR-3116**	2.50	0	**miR-3134**	-2.27	2
**miR-5689**	2.48	0	**miR-5190**	-2.27	1
**miR-608**	2.44	0	**miR-1285-5p**	-2.27	2
**miR-1249-5p**	2.38	0	**miR-550b-2-5p**	-2.23	0
**miR-6819-5p**	2.34	0	**miR-4480**	-2.20	1
**miR-4664-5p**	2.34	0	**miR-4703-5p**	-2.14	0
**miR-4514**	2.31	1	**miR-143-5p**	-2.09	1
**miR-8073**	2.27	0	**miR-519a-3o**	-2.02	1
**miR-4672**	2.26	1	**miR-181b-2-3p**	-2.01	0
**miR-4732-5p**	2.23	0			
**miR-593-5p**	2.21	0			
**miR-92a-1-5p**	2.17	0			
**miR-5589-5p**	2.17	0			
**miR-6804-3p**	2.15	0			
**miR-16-5p**	2.10	0			
**miR-3126-5p**	2.07	1			
**miR-3616-3p**	2.05	0			
**miR-3918**	2.05	0			
**miR-4688**	2.03	0			
**miR-6515-5p**	2.02	1			
**miR-1199-3p**	2.02	0			

Only miRNAs with a z-score > |2.0| are listed. The number of potential binding sites within the NT5E 3′-UTR were obtained by miRmap.

Focusing on miRNAs modulating NT5E expression in both SK-Mel-28 and MDA-MB-231 cells, we identified 16 miRNAs that significantly reduced NT5E surface expression in both cell lines, while 10 miRNAs induced increased NT5E expression (**Figure 1C**). No miRNA exerted opposing effects on the two cell lines. The list of all miRNAs that affected NT5E surface levels in the library screen is provided in the **Supplementary File**: NT5E_screen_miRNAs.xlsx.

Furthermore, our screen revealed 43 miRNAs that significantly lowered and 61 miRNAs that elevated ENTPD1 surface levels in SK-Mel-28 cells (**Supplementary Figure S3A**). The top three ENTPD1 enhancing miRNAs were miR-6733-3p (z-score = 3.5), miR-34b-3p (z-score = 3.2), and miR-208a-3p (z-score = 3.1). miR-6730-5p (z-score = -3.2), miR-5681a (z-score = -3.2), and miR-585-3p (z-score = -2.8) exerted the strongest inhibitory effects on ENTPD1 expression. Ten of the 43 ENTPD1-inhibitory miRNAs were predicted to target ENTPD1 by binding to its 3′-UTR, whereas only 4 of the enhancing miRNAs represented potential binders (p = 0.0194, two-sided Fisher’s exact test). Regarding miRNA-mediated modulation of CD274 expression levels, we found 107 miRNAs that significantly reduced CD274 surface levels (**Supplementary Figure S3B**). The strongest downregulation was measured after transfection with miR-512-3p (z-score = -3.9), miR-1273c (z-score = -3.8), and miR-1204 (z-score = -3.2). Overall, 130 miRNAs enhanced CD274 surface levels, with the strongest changes mediated by miR-3928-3p (z-score = 5.1), miR-5701 (z-score = 4.5), and miR-6513-3p (z-score = 4.2). Similar to NT5E, CD274-inhibiting miRNAs were enriched for miRNAs with predicted binding sites for the CD274 3′-UTR (p = 0.0035, two-sided Fisher’s exact test).

### 3.3 miRNA-mediated effects on NT5E surface expression are verified in various tumor cell lines

miRNAs showing significant effects on NT5E surface expression in both cell lines were selected for further validation (**Supplementary Table S9**). Additionally, miR-34b-3p was included because of its enhancing effect on NT5E and ENTPD1 expression in SK-Mel-28 cells (**Figure 1A**; **Supplementary Figure S3A**). miR-1293 and miR-3116 were selected for further testing, as both miRNAs enhanced the surface expression of NT5E and CD274 in MDA-MB-231 cells (**Figure 1B**; **Supplementary Figure S3B**). Since miR-193b-3p and miR-193a-3p share the same binding sites to the NT5E 3′-UTR, both miRNAs were included in further validation steps. All the miRNAs selected for further testing are listed in **Table 3**.

**Table 3:** List of selected miRNAs affecting NT5E surface expression in MDA-MB-231 and SK-Mel-28 cells.

SK28↑ & MDA ↑	BS	SK28 ↓ & MDA↓	BS
hsa-miR-16-5p	0	hsa-miR-1285-5p	2
hsa-miR-127-5p	1	hsa-miR-1298-3p	1
hsa-miR-1293*	0	hsa-miR-143-5p	1
hsa-miR-134-3p	0	hsa-miR-148b-3p	1
hsa-miR-224-3p	1	hsa-miR-155-5p	2
hsa-miR-3116*	0	hsa-miR-181b-2-3p	0
hsa-miR-3126-5p	1	hsa-miR-193a-3p	1
hsa-miR-34b-3p^†^	0	hsa-miR-193b-3p*	1
hsa-miR-4672	1	hsa-miR-22-3p	1
hsa-miR-6514-3p	0	hsa-miR-3118	2
hsa-miR-6859-3p	0	hsa-miR-3134	2
		hsa-miR-520d-3p	1

For each miRNA the number of binding sites (BS) within the NT5E 3′-UTR is given based on miRmap.

* Significant effect in MDA-MB-231 cells only, but same tendency in the SK-Mel-28 cell line.

**^†^** Significant effect in SK-Mel-28 cells only, but the same tendency in MDA-MB-231 cell line.

**Figure 2:**
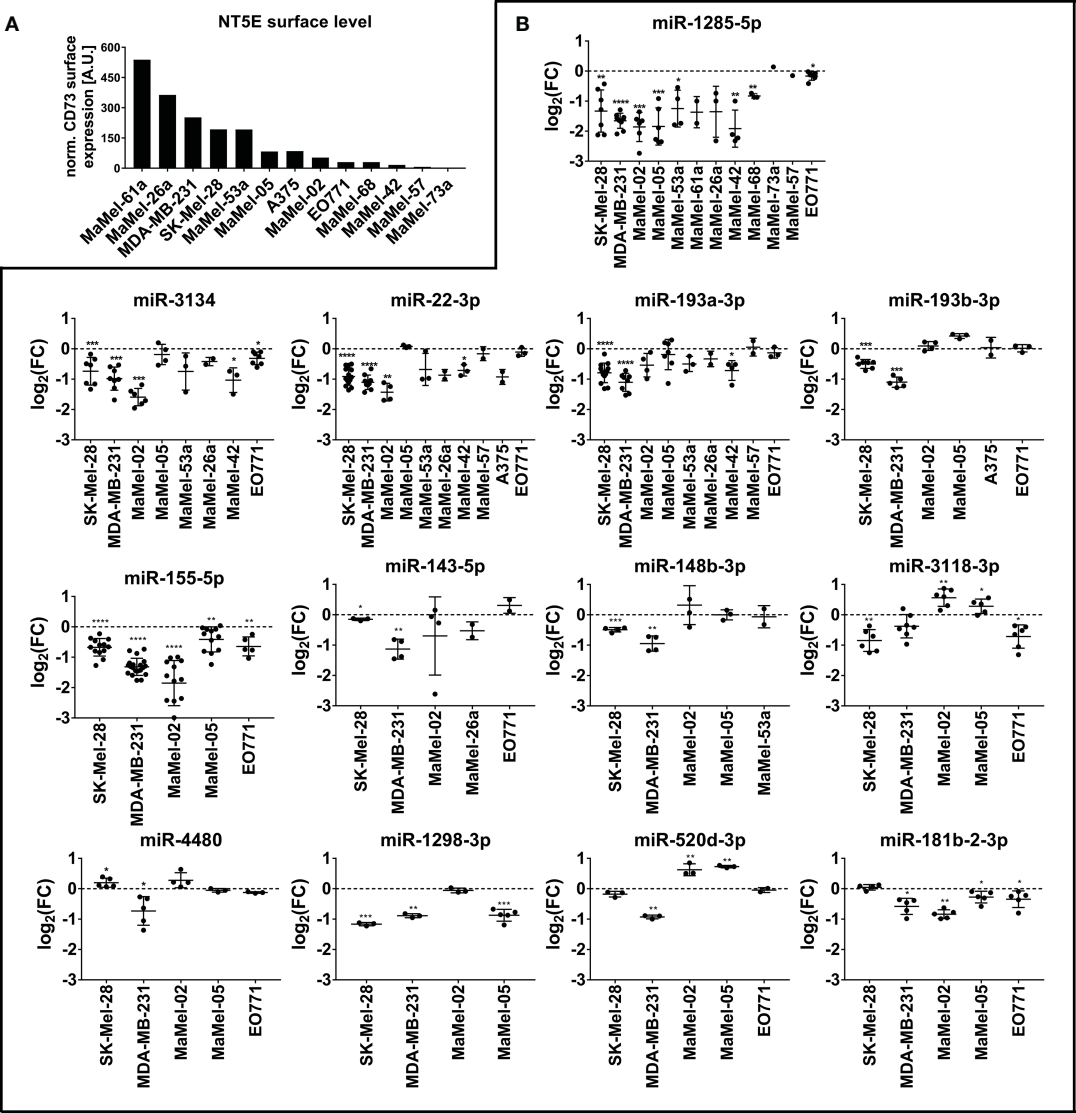
Validation of NT5E downregulating miRNAs by flow cytometry. **(A)** The basal NT5E surface expression level of cell lines used for the validation of the screen results are listed from high to low expressing cell lines. MFI values were normalized to the respective isotype control. **(B)** Effect of the selected miRNAs inhibiting NT5E surface expression was confirmed in independent transfection experiments. Each dot represents an independent experiment. Fold changes in MFI were calculated compared to the respective mimic control-1 samples. Mean ± SD are shown. Significance was assessed by one-sample T-test. *p < 0.05; **p < 0.01; ***p < 0.001; ****p < 0.0001.

Confirmatory transfection experiments using tumor cell lines with different basal NT5E expression levels (**Figure 2A**) showed that miR-1285-5p caused a strong reduction in NT5E surface expression in 10 out of the 12 cell lines tested, which is in line with the inhibitory effect on NT5E surface expression seen with this miRNA in the library screen (**Figure 2B**). Only MaMel-73a and MaMel-57, which exhibited very low basal expression levels of NT5E, failed to show reduced NT5E expression. A similar result was observed for miR-22-3p, which induced consistent downregulation of NT5E surface levels in 7 out of 10 cell lines, reminiscent of the inhibitory effect observed with this miRNA on the screen. The screening results were also confirmed for miR-193a-3p and miR-193b-3p, which showed a significant reduction in NT5E expression in SK-Mel-28 and MDA-MB-231 cells. Moreover, miR-143-5p and miR-148b-3p significantly reduced NT5E surface levels in these cell lines, confirming the screening results. However, the effect on the other tested cell lines was only minor or even inverse. The inhibitory effect of miR-3118-3p on NT5E expression was confirmed in the SK-Mel-28 cell line, however in MDA-MB-231 cells this effect was insignificant. Moreover, MaMel-02 and MaMel-05 showed increased NT5E levels after miR-3118-3p transfection. NT5E surface expression was significantly reduced in SK-Mel-28, MDA-MB-231, and MaMel-05 cells following miR-1298-3p transfection. Similarly, miR-155-5p lowered NT5E surface expression in SK-Mel-28, MDA-MB-231, MaMel-02, and MaMel-05 cell lines. The inhibitory effect of miR-181b-2-3p on NT5E expression could only be validated in the MDA-MB-231 and MaMel-02 cell lines, whereas in SK-Mel-28 and MaMel-05 cells, this miRNA had no effect on NT5E surface expression levels. The inhibitory effect of miR-520d-3p was confirmed only in MDA-MB-231 cells, whereas MaMel-02 and MaMel-05 cells showed enhanced NT5E surface expression upon miR-520d-3p transfection.

**Figure 3:**
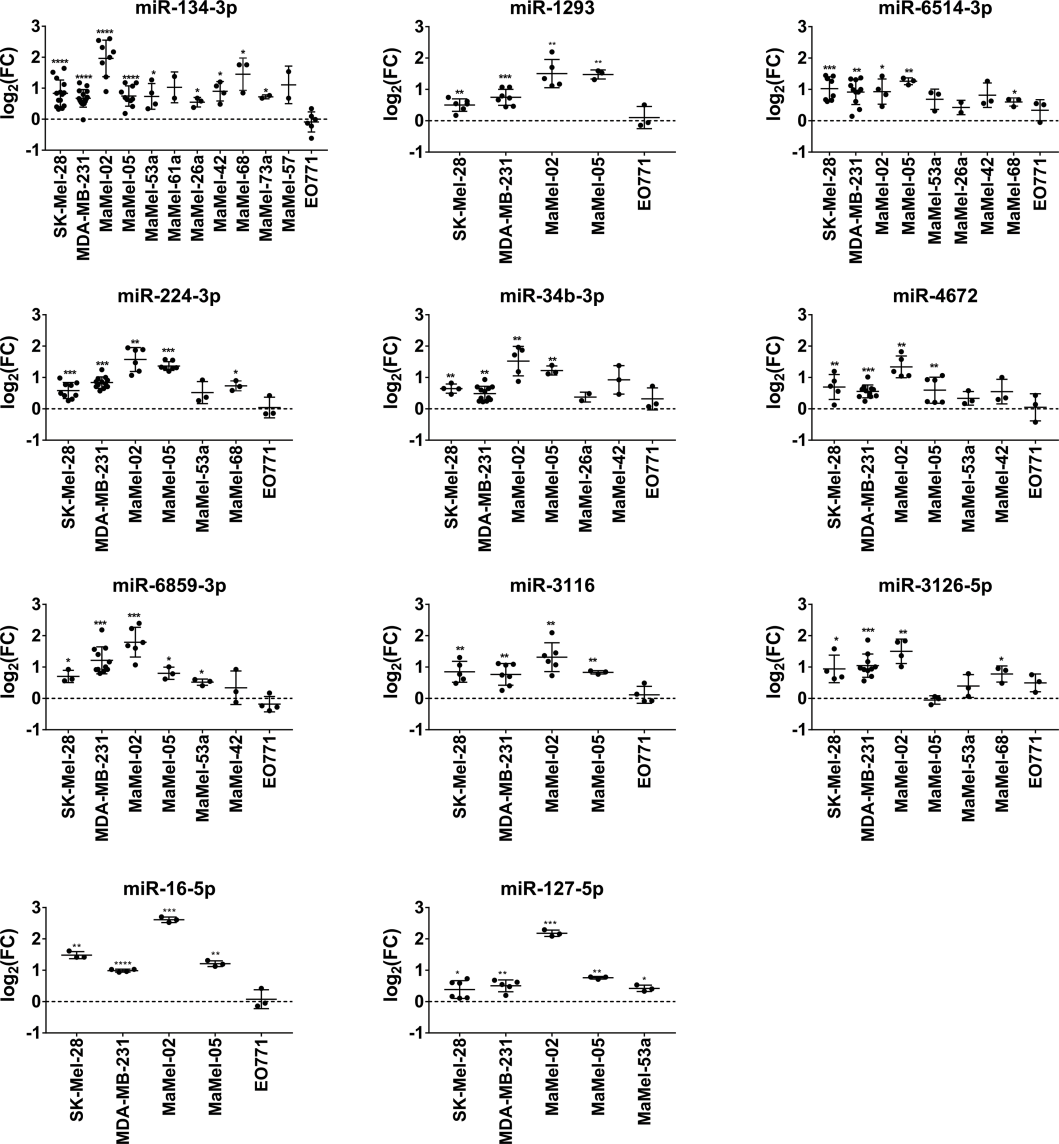
Validation of NT5E upregulating miRNAs by flow cytometry. The effect of the selected NT5E enhancing miRNAs from the library screen was confirmed in independent transfection experiments. Each dot represents an independent experiment. Fold changes in MFI were calculated compared to the respective mimic control-1 samples. Mean ± SD are shown. Significance was assessed by one-sample T-test. *p < 0.05; **p < 0.01; ***p < 0.001; ****p < 0.0001.

Regarding miRNAs with an enhancing effect on NT5E expression, we verified the screening results for all the miRNAs selected. Notably, the activating capacities of these miRNAs were consistent across the tested cell lines. In fact, miR-134-3p, representing the strongest hit in the library screen of SK-Mel-28 cells, consistently enhanced NT5E surface levels in all 12 tested cell lines. Moreover, a significant increase in NT5E expression was observed in nine cell lines (**Figure 3**). Furthermore, miR-6514-3p, miR-6859-3p, and miR-224-3p upregulated NT5E surface levels in majority of tested cell lines, and this increase was significant in five cell lines. We observed significantly enhanced NT5E surface levels upon transfection with miR-3126-5p or miR-3116 in the three tumor cell lines SK-Mel-28, MDA-MB-231, and MaMel-02. miR-4672, miR-1293, and miR-34b-3p significantly increased NT5E surface expression in the SK-Mel-28, MDA-MB-231, MaMel-02, and MaMel-05 cell lines. miR-127-5p significantly enhanced NT5E surface levels in all five tested cell lines, with the strongest effects observed in MaMel-02 cells. Furthermore, miR-16-5p strongly enhanced NT5E surface expression in all five cell lines tested.

### 3.4 miRNA-mediated effects on NT5E surface expression are verified at the transcriptional level

**Figure 4:**
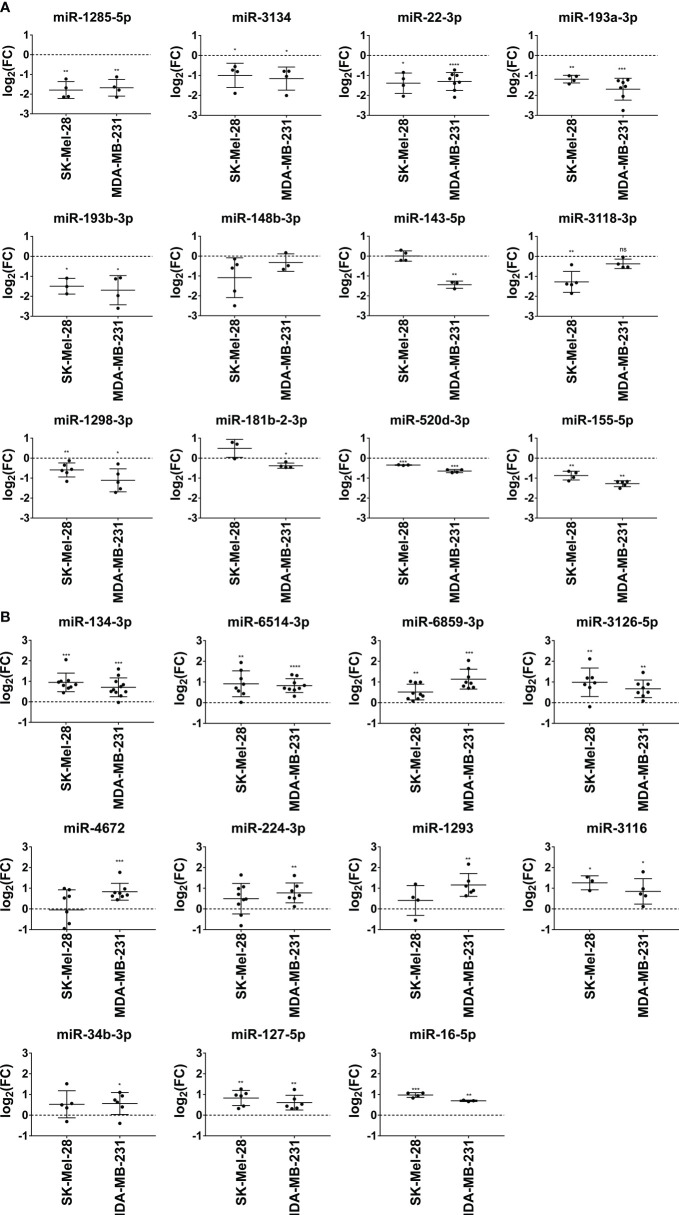
Validation of miRNAs modulating NT5E expression by qPCR. The effect of the selected NT5E inhibiting miRNAs **(A)** and NT5E enhancing miRNAs **(B)** from the library screen was confirmed in independent transfection experiments. Each dot represents one experiment. Cell lines were transfected with 50 nM miRNA. NT5E mRNA levels were determined by qPCR 48 h post transfection. Fold changes were calculated compared to the respective mimic control-1 samples. RPL19 was used as the housekeeping gene. Mean ± SD are shown. Significance was assessed by one-sample T-test. *p < 0.05; **p < 0.01; ***p < 0.001; ****p < 0.0001.

Next, we examined the modulating effects of the selected miRNAs at the transcriptional level. We observed that miR-1285-5p, miR-1298-3p, miR-3134, miR-22-3p, miR-193a/b-3p, miR-520d-3p, and miR-155-5p significantly reduced NT5E mRNA expression in SK-Mel-28 and MDA-MB-231 cells (**Figure 4A**). The inhibitory effect of miR-143-5p on NT5E mRNA expression was more pronounced in the MDA-MB-231 cell line, whereas SK-Mel-28 cells showed no change in NT5E mRNA levels, reminiscent of the validation results obtained by the flow cytometry analysis described above. miR-148b-3p induced a trend towards reduced NT5E mRNA levels in SK-Mel-28 cells but not in the MDA-MB-231 cell line. Similarly, miR-3118-3p significantly lowered NT5E mRNA levels in SK-Mel-28 cells but not in the MDA-MB-231 cell line. In contrast, miR-181b-2-3p significantly reduced NT5E mRNA expression in MDA-MB-231 cells, whereas no significant effect was observed in the SK-Mel-28 cell line.

The majority of miRNAs that upregulated NT5E surface expression also increased NT5E mRNA levels. Thus, miR-134-3p, miR-3126-5p, miR-6859-3p, miR-6514-3p, miR-16-5p, and miR-3116 led to a significant upregulation of NT5E mRNA levels in both SK-Mel-28 and MDA-MB-231 cells (**Figure 4B**). miR-4672 induced significant upregulation of NT5E mRNA expression in MDA-MB-231 cells, while SK-Mel-28 cells showed variable effects. NT5E mRNA levels were also significantly enhanced upon transfection with miR-224-3p or miR-34b-3p in MDA-MB-231 cells, and the same trend was observed in SK-Mel-28 cells. Transfection with miR-127-5p significantly enhanced NT5E mRNA expression in both the tumor cell lines.

Based on the results obtained thus far, miR-3118-3p, miR-143-5p, miR-520d-3p, and miR-181b-2-3p were excluded from further investigation because they yielded inconsistent results during the preceding validation steps.

### 3.5 miR-1285-5p induced inhibition of NT5E expression is mediated by direct interaction with the NT5E 3′-UTR

**Figure 5:**
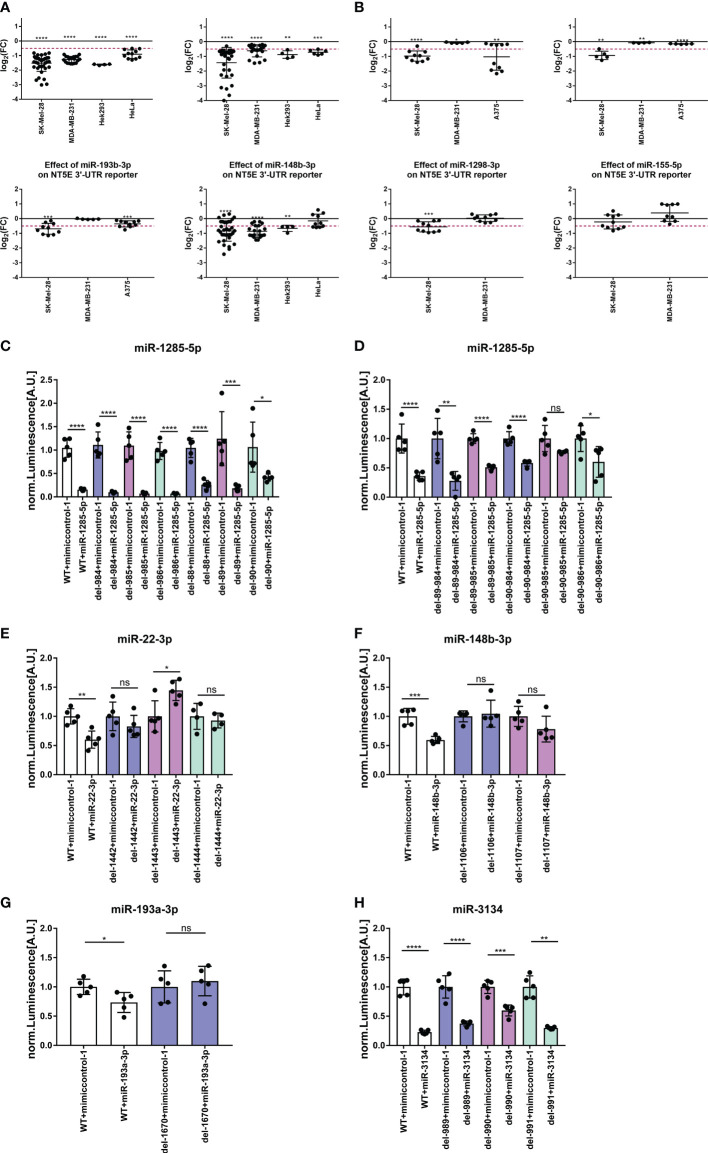
NT5E-3′-UTR reporter assay. The capacity of selected NT5E inhibiting miRNAs for direct NT5E 3′-UTR interaction was determined in luciferase reporter assays. Cells were transfected with 25 nM miRNA in a 96-well format, and luciferase activity was measured 24 h later. The effect of miRNAs on luminescence signal intensity with wild-type NT5E 3′-UTR is given in **(A)**. Each dot represents an individual transfection. Fold changes in luminescence signal intensity was calculated compared to the respective mimic control-1 samples. Significance was assessed by one-sample T-test. Effect of site-specific deletions within the respective miRNA binding site was assessed by luciferase reporter assay **(B-G)**. Therefore, SK-Mel-28 cells were transfected with 25 nM miRNAs in a 96-well format. 24 h post transfection luminescence signal intensity was measured. Each dot represents an individual transfection. Luminesce signal was normalized to the level of the control transfections. Mean ± SD are shown. Significance was assessed by unpaired T-test. *p < 0.05; **p < 0.01; ***p < 0.001; ****p < 0.0001.

Next, we further validated the identified miRNAs predicted to encounter at least one binding site within the NT5E 3′ UTR by testing their capacity for direct NT5E 3′-UTR interaction. Luciferase-based reporter assays performed on SK-Mel-28, MDA-MB-231, HEK293, and HeLa cells revealed the most pronounced inhibitory effects of miR-1285-5p, which lowered the luminescence signal in all four cell lines tested (**Figure 5A**). This is in line with the inhibitory effects on NT5E expression observed with miR-1285-5p on the protein and transcriptional levels in various tumor cell lines (**Figures 2**, **Figure 3A**). Similarly, miR-3134 significantly reduced luciferase activity in all four cell lines, while miR-148b-3p lowered the luminescence signal in three of the four cell lines tested. A significant reduction in the luminescence signal was also observed with miR-22-3p, miR-193a/b-3p, and miR-1298. These effects were most pronounced in SK-Mel-28 cells but were much less prominent in MDA-MB-231 cells. miR-155-5p showed no significant effect on the NT5E 3′-UTR reporter assays performed in the two cell lines.

We then introduced binding site-specific single nucleotide deletions in the NT5E 3′-UTR of the reporter plasmid within the seed sequences of miR-1285-5p, miR-3134, miR-148b-3p, miR-22-3p, and miR-193a-3p to prove the direct binding of these miRNAs to the NT5E 3′-UTR (**Table S10**). The deletion of a nucleotide within the contact sites is expected to disrupt the binding of the miRNA to the 3′-UTR, resulting in a diminished luminescence signal intensity. NT5E 3′-UTR contains two binding sites for miR-1285-5p. In our assays, the disruption of one binding site failed to restore the luminescence signal (**Figure 5B**). However, deletion at position 90 (del90) partially restored the luciferase activity. Thus, we generated constructs with single-nucleotide deletions in each of the two miR-1285-5p binding sites (**Figure 5C**), resulting in restored luciferase activity. Deletions at positions 90 and 985 (del90-985) restored the luminescence signal to the level of the control transfection. miR-22-3p encounters one binding site within the NT5E 3′-UTR, and single bp deletions at positions 1442, 1443, or 1444 completely rescued luciferase activity (**Figure 5D**). Notably, deletion at position 1443 increased the luminescence signal, beyond that of the control level. Similarly, the NT5E 3′-UTR was predicted to bear one binding site for miR-193a-3p, and a bp deletion at 1670 rescued the luminescence signal to that of the control level (**Figure 5E**). Finally, the NT5E 3′-UTR contained two putative binding sites for miR-3134; however, for technical reasons, constructs with deletions at the first binding site (positions 352, 353, and 354) could not be generated. Single-bp deletions in the second binding site partially restored the luminescence signal, which was most pronounced upon bp deletion at position 990 (**Figure 5F**). Thus, we suspect that disruption of both binding sites is mandatory to fully abrogate miR-3134 mediated inhibition of luciferase expression. Moreover, the NT5E 3′-UTR harbors one binding site for miR-148b-3p; consequently, a bp deletion at position 1106 or 1107 within the NT5E 3′-UTR impeded miR-148b-3p mediated inhibition of luciferase activity compared to that in the wild-type 3′-UTR (**Figure 5G**).

### 3.6 miRNAs enhance NT5E expression through direct and indirect mechanisms

Notably, miR-127-5p, miR-224-3p, miR-3126-5p, and miR-4672 mediated enhanced NT5E surface expression, as shown in **Figure 4**, and were also predicted by the miRmap tool as potential NT5E 3′-UTR binders. Thus, luciferase reporter assays were performed using these miRNAs in various tumor cell lines (**Supplementary Figure S4**). miR-4672 reduced the luciferase signal in all four cell lines tested, indicating direct binding to the NT5E 3′-UTR and mRNA destabilization. miR-224-3p and miR-3126-5p lowered the luciferase signal in three of the four and two of the four cell lines, respectively. Notably, miR-127-5p significantly enhanced the luciferase reporter signal in both the cell lines. Thus, we suspect that the enhancing effects on NT5E expression are mediated by miR-224-3p, miR-3126-5p, and miR-4672 through indirect mechanisms, whereas miR-127-5p acts through direct interaction with the NT5E 3′-UTR with an unknown mechanism of upregulation by binding to the 3′-UTR.

### 3.7 Indirect miRNA-mediated upregulation of NT5E expression correlates with the reduced expression levels of predicted NT5E repressors

Next, we intended to uncover the possible mechanisms responsible for the miRNA-mediated enhancement of NT5E surface expression in SK-Mel-28, MaMel-02, and MDA-MB-231 cells. Thus, we combined correlation analysis, miRNA target prediction, and microarray analysis after miRNA transfection of these cell lines, and analyzed the resulting effects on global gene expression caused by the respective miRNAs (**Supplementary Table S11**). Since we hypothesized that these miRNAs might indirectly enhance NT5E surface levels by blocking one or several NT5E repressors, we used RNA sequencing data from ten melanoma cell lines and five normal human melanocyte samples to obtain a list of genes that showed a significant negative correlation with NT5E mRNA expression (**Supplementary Excel File** NT5E_Cor_RNASeq.xlsx). Additionally, we focused on genes whose mRNAs were predicted to harbor binding sites within their 3′-UTR for NT5E enhancing miRNAs.

**Figure 6:**
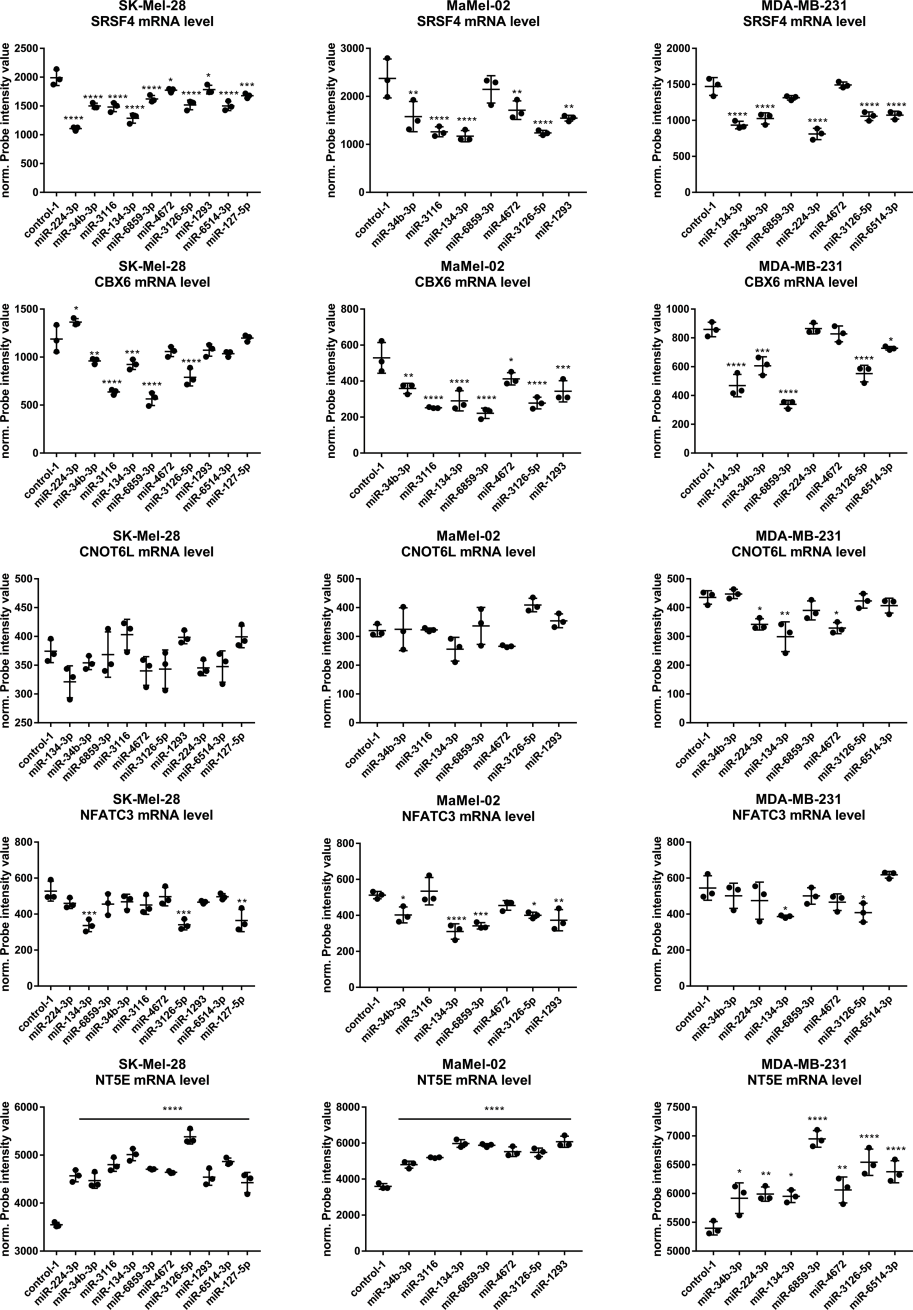
miRNA mediated upregulation of NT5E surface expression correlates with reduced expression levels of the predicted NT5E repressors. Cancer cells were transfected with 50 nM miRNA, and cells were harvested 48 h after treatment. Triplicates were performed per condition. RNA was isolated and used for microarray profiling. Changes in expression levels of CBX6, CNOT6L, NFATC3, NT5E, and SRSF4 levels are depicted. Mean ± SD are shown. Significance was assessed by One-way Anova with Dunnett’s multiple comparison *p < 0.05; **p < 0.01; ***p < 0.001; ****p < 0.0001.

Following this approach, we identified chromobox 6 (CBX6) and CCR4-NOT transcription complex subunit 6 (CNOT6L) as promising candidates. CBX6 is involved in the maintenance of transcriptional repression through epigenetic modifications (33, 34). Moreover, CBX6 expression is negatively associated with NT5E mRNA levels (PCC = -0.53, p = 0.043); in fact, ten NT5E enhancing miRNAs shown in **Table S11** had at least one predicted binding site for CBX6 3′-UTR (**Table S12**). CNOT6L is a catalytic component of mRNA de-adenylase CCR4-NOT, which is involved in the miRNA-mediated repression of translation (35, 36) (PCC = -0.57, p = 0.027). Notably, eight of the NTE5 enhancing miRNAs were predicted to bind to the 3′-UTR of CNOT6L. Based on the microarray data, we determined which miRNA could lower the expression of putative NT5E inhibitors and found that serine- and arginine-rich splicing factor 4 (SRSF4) was downregulated upon transfection of miR-134-3p or miR-224-3p in all tested cell lines (**Figure 6**). Snider et al. showed that NT5E has two isoforms in humans and that the short NT5E variant can inhibit the longer variant, thus reducing the NT5E surface levels (37). Overall, four NT5E enhancing miRNAs contained at least one predicted binding site in the SRSF4 3′-UTR (**Table S12**). Additionally, we found that nuclear factor of activated T cells 3 (NFATC3) was significantly lowered upon miR-134-3p transfection in all three cell lines (**Figure 6D**). NFATC3 mRNA expression showed a significant negative correlation with NT5E mRNA levels in the NCI-60 dataset (PCC = -0.573). NFATC3 was predicted to bind to the NT5E promoter region and might thus act as a transcriptional repressor of NT5E. Seven of the NT5E enhancing miRNAs had at least one predicted binding site for the NFATC3 3′-UTR. Therefore, we also included SRSF4 and NFATC3 in subsequent analyses.

Having confirmed that NT5E enhancing miRNAs can modulate the expression of the potential NT5E repressors shown above, we proceeded with individual siRNA knockdowns of the respective targets.

### 3.8 siRNA-mediated knock down of uncovered NT5E repressors recapitulates miRNA induced amplification of NT5E expression

**Figure 7:**
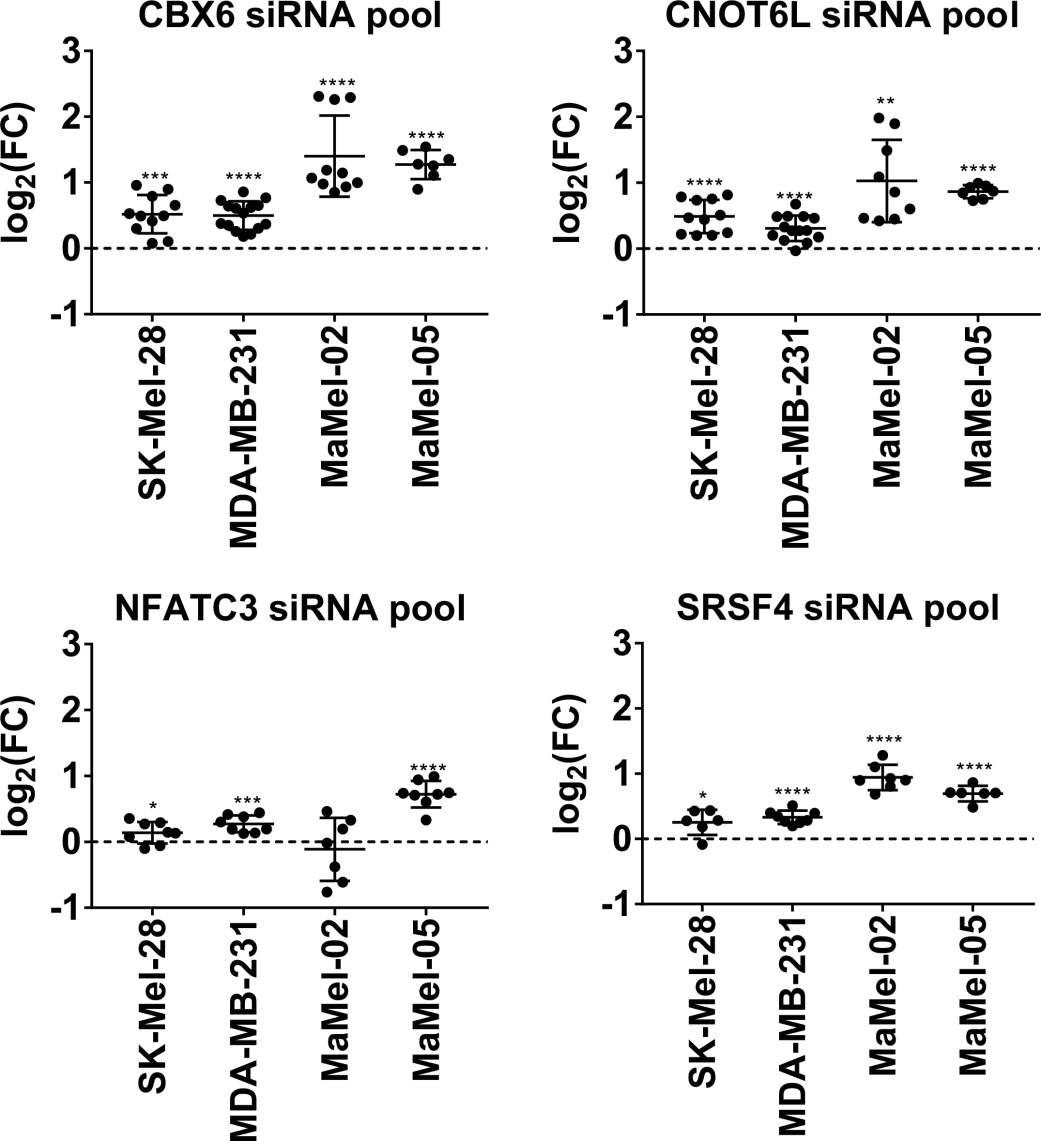
siRNA-mediated knock down of uncovered NT5E repressors recapitulates miRNA-induced amplification of NT5E expression. The effect of the selected NT5E repressors that may mediate miRNA-induced NT5E upregulation was confirmed in independent transfection experiments. Each dot represents an independent experiment. Fold changes in MFIs were calculated compared to the respective mimic control-1 samples. Mean ± SD are shown. Significance was assessed by one-sample T-test. *p < 0.05; **p < 0.01; ***p < 0.001; ****p < 0.0001.

In the following validation step, we tested whether individual knockdown of the identified miRNA targets by siRNA-mediated silencing could recapitulate the upregulated NT5E surface expression observed with the NT5E inducing miRNAs. As shown in **Figure 7**, the knockdown of CBX6, CNOT6L, SRSF4, or NFACT3 increased the surface expression of NT5E in all tested cell lines. This increase was less pronounced in the two breast cancer cell lines, SK-Mel-23 and MDA-MB-231, than that in the two melanoma cell lines, and NFACT3 knockdown failed to induce NT5E upregulation in MaMel-02 cells. Overall, siRNA-mediated knockdown of CBX6, CNOT6L, and SRSF4 consistently enhanced NT5E surface levels in various human tumor cell lines.

Next, we compared the dynamic and steady upregulation of NT5E surface expression mediated by siRNAs and miRNAs (**Supplementary Figure S5**). Therefore, we selected miR-134-3p and miR-224-3p, as shown in **Figure 6**, to inhibit the expression of both CBX6 and CNOT6L or of CNOT6L only, respectively, in SK-Mel-28 cells, including miR-6514-3p as a negative control (**Figure 6**). Following the transfection of SK-Mel-28 cells with the miRNAs or siRNAs mentioned above, miRNA-mediated upregulation of NT5E expression proceeded faster than the siRNA-induced increase in NT5E surface expression (**Supplementary Figure S5A**), and the enhancing effect reached higher levels with miR-134-3p and miR-6514-3p than that with CBX6 or CNOT6L siRNA-mediated knockdown (**Supplementary Figure S5B**). Remarkably, at the 72-h time point, miR-224-3p transfection resulted in upregulated NT5E levels similar to those observed upon siRNA-mediated silencing of CBX6 and CNOT6L expression. Notably, co-transfection of miR-224-3p with either CBX6 or CNOT6L siRNA did not further increase NT5E expression. In addition, the combined transfection of CBX6 and CNOT6L siRNA failed to improve NT5E upregulation, indicating a redundant mode of action. We conclude that indirect upregulation of NT5E by miR-224-3p is most likely caused by CNOT6L inhibition, while the enhancing effect of miR-134-3p on NTE expression can only be partially explained by miR-134-3p dependent inhibitory effects on CBX6 and CNOT6L. The miR-6514-3p–mediated increase in NT5E expression is likely caused by unknown mechanisms.

### 3.9 miR-6859-3p- and miR-6514-3p-mediated enhancement of NT5E expression depends on proteasomal activity

From the microarray data (**Supplemental File 2**), we noticed that each NT5E enhancing miRNA caused a unique transcriptomic profile change, with few similarities among these miRNAs. We then performed NT5E-promoter reporter assays and conducted analyses with the proteasome inhibitor lactacystin (38) to unravel the underlying mechanisms of miRNA-mediated NT5E upregulation. NT5E promoter assays showed no changes in luciferase activity upon miRNA transfection, indicating that no direct transcriptional repressor was inhibited by these miRNAs (**Supplementary Figure S6**) (38). Treatment with 5 µM lactacystin only had a marginal impact on the NTE surface expression levels in untreated cells or those transfected with the control miRNA (**Supplementary Figure S7A**). Notably, treatment of cells with lactacystin completely abolished the NT5E increase in miR-6859-3p transfected MDA-MB-231 cells. Similarly, the NT5E enhancing effect of miR-6514-3p was reduced upon proteasomal inhibition. Regarding the remaining miRNAs, lactacystin treatment did not affect the miRNA-induced upregulation of NT5E surface levels, indicating that miR-6859-3p and miR-6514-3p mediated enhancement of NT5E expression is dependent on proteasomal function. Based on our microarray data, transfection of miR-6514-3p significantly inhibited AGO2 mRNA levels in MDA-MB-231 cells (FC = 0.59, p < 0.0001) and SK-Mel-28 cells (FC = 0.75, p < 0.01), as well as decreased the levels of DROSHA (FC = 0.67, p = < 0.0001; FC = 0.79, p < 0.01), which represent essential gene products of the miRNA biogenesis pathway. We speculated that part of the NT5E activation by miR-6514-3p is mediated via intrinsic changes in intracellular miRNA levels. Thus, we measured the intracellular levels of miR-22-3p, miR-193a-3p, and miR-1285-5p, which were found to suppress NT5E expression in MDA-MB-231 cells (**Figure 2**) after transfection with miR-6514-3p (**Supplementary Figure S8**). Indeed, miR-22-3p and miR-193a-3p expression levels significantly decreased following miR-6514-3p transfection.

### 3.10 miR-6859-3p and miR-134-3p impacts occurrence of NT5E splicing variants

As mentioned above, there are two isoforms of human NT5E, and the shorter isoform can bind to the long isoform, thereby causing its proteasomal degradation (37). Hence, we reused the samples bound for microarray analysis and quantified the isoform-specific effects of miRNAs by qPCR in MDA-MB-231 and SK-Mel-28 cells. We calculated the fold changes in isoform expression following transfection with NT5E enhancing miRNAs (**Supplementary Figure S9**). If NT5E splicing is unaffected by miRNAs, equal induction of both variants would be expected, resulting in a delta value of both fold changes close to zero. This applies to miR-4672, miR-6514-3p, miR-127-5p, and miR-3116 in SK-Mel-28 cells, and miR-4672 in MDA-MB-231 cells. However, for miR-6859-3p, we observed a significant difference in SK-Mel-28 cells, with the same tendency in MDA-MB-231 cells. Regarding miR-134-3p, a significant effect was observed in MDA-MB-231 cells, with the same tendency as in SK-Mel-28. This implies that miR-134-3p and miR-6859-3p lead to a higher rate of upregulation of the long NT5E isoform than that of the shorter variant. Especially for miR-6859-3p in SK-Mel-28 cells, we observed no ΔCt change in NT5E-2 expression compared to mimic control-1 samples, whereas ΔCt of NT5E-1 dropped significantly.

We also tested the effect of miR-134-3p on isoforms in SK-Mel-28 and MaMel-05 cells (**Supplementary Figure S10**) and found opposing effects of miR-134-3p on the expression levels of the two NT5E isoforms, which was most pronounced in MaMel-05 cells. While the normal NT5E isoform level increased to the expected extent (log_2_FC = 1.5), a strong decrease in the shorter NT5E isoform level was measured (log_2_FC = -20.5). In SK-Mel-28 cells, miR-134-3p had a strong impact on the normal isoform. Notably, the NT5E siRNA pool uniformly inhibited both NT5E isoforms to the same extent.

### 3.11 Effect of miRNAs on NT5E mediated AMP hydrolysis

Immunosuppressive adenosine generated through NT5E mediated AMP hydrolysis helps cancer cells escape their elimination by activated CTL (23). To assess whether the miRNAs found to modulate NT5E expression levels in human cancer cells would also affect AMP turnover, we applied the malachite green assay, as described by Allard et al. (31), to four cell lines exhibiting different levels of basal AMP turnover.

**Figure 8:**
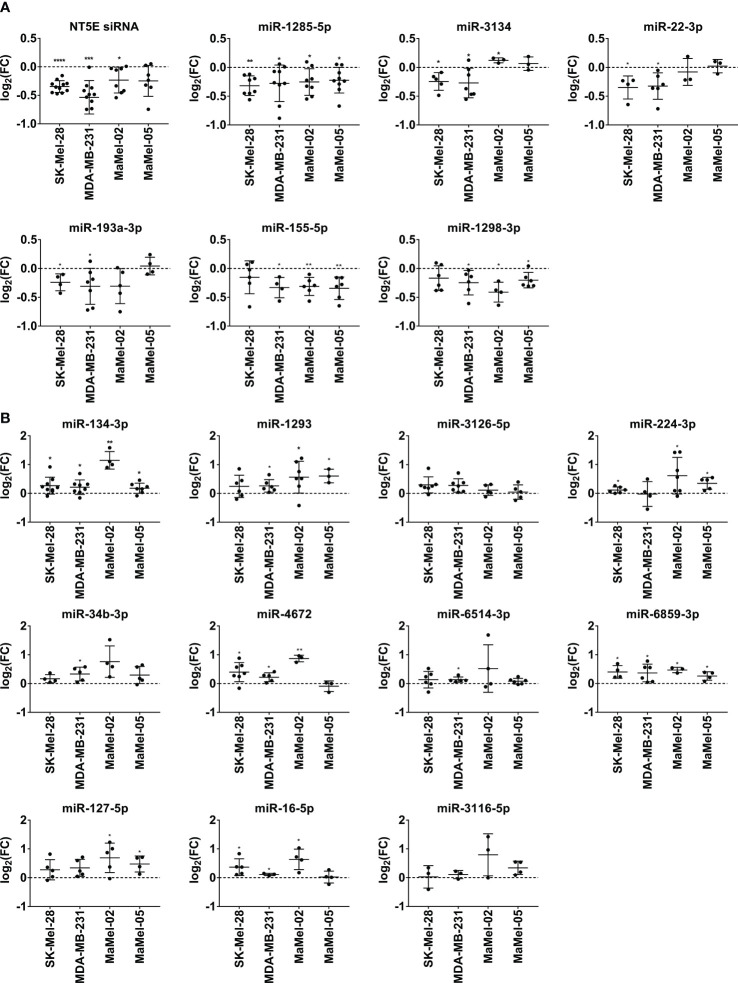
Effect of miRNAs on AMP turnover mediated by NT5E. Cancer cells were transfected with 50 nM miRNA/siRNA. **(A)** For NT5E-inhibiting miRNAs 400 µM AMP was added to the transfected cells 48 h post transfection. **(B)** For NT5E-enhancing miRNAs 400 µM AMP was added 72 h post transfection. Supernatant was collected 30 min after AMP supplementation, and the amount of released phosphate was measured using malachite green assay. Technical replicates (5–8) were performed per condition and assay. Fold changes were calculated to mimic the control-1 condition of the respective experiment. Each dot represents an independent experiment. Mean ± SD are shown. Significance was assessed by one-sample T-test. *p < 0.05; **p < 0.01; ***p < 0.001; ****p < 0.0001.

Overall, all the selected miRNAs were capable of reducing the enzymatic activity of NT5E (**Figure 8A**). As seen in the transfection experiments and reporter assays described above, miR-1285-5p showed the strongest and most consistent effects in this functional assay, significantly lowering AMP turnover in all the four tested cell lines. miR-155-5p and miR-1298 had significant effects in MDA-MB-231, MaMel-02, and MaMel-05 cells. miR-3134, miR-22-3p, and miR-193a-3p decreased NT5E enzymatic activity in SK-Mel-28 and MDA-MB-231 cell lines. Regarding NT5E enhancing miRNAs (**Figure 8B**), miR-134-3p and miR-6859-3p led to the most uniform increase in AMP turnover, with a significant increase in AMP levels in all four cell lines. miR-4672 and miR-16-5p significantly upregulated NT5E enzymatic activity in all three cell lines, except for MaMel-05. miR-1293 had significant effects on MDA-MB-231 and MaMel-05 cells, and the same tendency was observed in MaMel-02 and SK-Mel-28 cells. Similarly, miR-127-5p increased AMP turnover in MaMel-02 and MaMel-05 cells, and the same trend was observed in MDA-MB-231 and SK-Mel-28 cell lines. Moreover, miR-224-3p enhanced AMP concentrations in SK-Mel-28 and MaMel-05 cells, whereas transfection of miR-3126-5p significantly increased phosphate generation in SK-Mel-28 and MDA-MB-231 cells. We found that miR-6514-3p, miR-34b-3p, and miR-3116 enhanced NT5E enzymatic activity; however, this effect was restricted to MaMel-05 cells.

## 4 Discussion

Based on our miRNA library screening, we identified a panel of ICMs whose expression is modulated by miRNAs. In contrast to the stand-alone *in silico* prediction, the screening revealed miRNAs capable of reducing or enhancing the expression of ICMs. The top candidates miR-422a, miR-155, and miR-193a/b predicted *in silico* were partially verified by our library screen, and the inhibitory effect of miR-155-5p on NT5E and CD274 expression was confirmed by screening and validated for NT5E. For miR-193a/b, we also confirmed the direct downregulation of NT5E by these miRNAs, although no effects on CD274 or ENTPD1 surface expression were measured during the screening. miR-422a has been reported to directly target the NT5E 3′-UTR (39), but within the library screen, we could not observe any significant effect on NT5E expression with this miRNA. It is possible that the miR-422a-mediated effect is cancer type-specific or that changes mediated by this miRNA are not always reflected in the NT5E surface level. This highlights that pure *in silico* predictions remain speculative, while the library screen allowed us to determine miRNAs that modulate immune checkpoint molecule expression at the cell surface level of tumor cells. Only changes at the surface level would have effects on the tumor microenvironment and could alter the susceptibility of cancer cells to killing by CTLs.

We found miRNAs that drastically and consistently lowered the NT5E surface expression. The most pronounced effects were mediated by miR-1285-5p and miR-3134 expression. Thus, we consider these miRNAs to be potential tumor-suppressive miRNAs. Indeed, several studies have described miR-1285-5p as a tsmiR. For instance, in renal cell carcinoma, miR-1285 expression is significantly downregulated compared to adjacent tissues, and re-expression of this miRNA reduces cancer cell migration, invasion, and proliferation. Hidaka et al. also performed microarray analysis after miR-1285 transfection and found 17 genes to be differentially downregulated. Notably, NT5E was one of these genes, which is in line with our findings (40). Furthermore, Hironaka-Mitsuhashi et al. demonstrated that low miR-1285-5p levels are correlated with poor prognosis in breast cancer, and re-expression of this miRNA in breast cancer cells inhibits proliferation by directly targeting TMEM194A (41). Although our work and previous studies would classify miR-1285-5p as a tsmiR for breast cancer, melanoma, pancreatic cancer, and renal cell carcinoma (40–42), this miRNA might act as an oncomiR in non-small cell lung cancer (NSCLC). Zhou et al. found that blocking miR-1285-5p with an antagomiR inhibits proliferation and metastasis of NSCLC cells (43).

Here, we describe for the first time miR-3134 as a potential tsmiR that drastically lowers the expression of NT5E in breast cancer and melanoma cells. To date, only one study has been published on the function of miR-3134 demonstrating the interplay between miR-3134 AU-rich element-mediated degradation (44). AU-rich elements are regulatory elements within the 3′-UTR, which are normally associated with AU-rich element-binding proteins, leading to destabilization of the respective mRNA molecule and its degradation (45). In the breast cancer cell line MCF7 Sharma et al. proved that miR-3134 overexpression increased the mRNA levels of several target genes such as SOX9, EGFR, VEGFA, and HLA-G. Of the genes that were upregulated upon miR-3134 overexpression, several genes encode major histocompatibility complex molecules (HLA) such as HLA-A, HLA-B, HLA-H, HLA-F, HLA-G, HLA-DRA, and HLA-DPA1 (44). This might be a very notable fact to keep in mind for further evaluation of this miRNA as a potential therapeutic agent. miR-3134 not only decreases NT5E levels, but also enhances expression of HLA molecules. Both mechanisms facilitate the CTL-mediated killing of cancer cells.

For most NT5E-inhibitory miRNAs, we demonstrated direct miRNA binding to the NT5E 3′-UTR as a mode of action. In addition to miRNAs inhibiting the expression of ICMs, our screen also identified several miRNAs that boosted ICM expression in cancer cells. These miRNAs may represent potential oncomiRs that facilitate tumor immune escape. Uncovering the underlying mechanisms of the observed NT5E upregulation may provide new options to counteract tumor immune escape. We identified several targets accounting for, at least in part, the miRNA-mediated enhancement of NT5E surface expression. Thus, CBX6 and CNOT6L were identified as prominent direct targets of NT5E enhancing miRNAs and notably, siRNA-mediated knockdown of these targets could mimic the observed increase in NT5E expression. Therefore, these genes inhibit NT5E expression under physiological conditions; hence, their downregulation might represent an escape mechanism that promotes cancer cell immune evasion. Indeed, high expression of NFATC3, CBX6 and CNOT6L is associated with better survival in breast cancer patients (**Supplementary Figure S11**). In accordance, high NT5E/CD73 expression level is significantly associated with worse prognosis for breast cancer patients. This indicates, that high levels of NT5E promoting miRNAs such as miR-224 or low expression of NT5E repressor such as CBX6/CNOT6L or NFACT3 are linked to progressive tumor disease reflected by shorter survival time. Furthermore, CBX6 has been described as a tumor suppressor in breast cancer (46) and mesothelioma (47). In addition to these direct targets, we found that proteasomal function was detrimental to NT5E upregulation by miR-6859-3p and miR-6514-3p. Furthermore, miR-6514-3p modulates NT5E levels by altering the intracellular miRNA expression profile. These two miRNAs have rarely been described in the literature, and we are the first to connect these miRNAs to the expression of ICMs. Notably, a recent study by Fernandez et al. found that miR-6514-3p is upregulated in prostate cancer patients, indicating its potential role as a prognostic marker in prostate cancer (48). Among the miRNAs described in our study, miR-134-3p is of particular interest because it enhanced the expression of all three ICMs investigated, that is, ENTPD1, NT5E, and CD274. Several studies have described miR-134 as an oncomiR in lung cancer (49), uveal melanoma (50), prostate cancer (51), and colon cancer (52), consistent with our data. This miRNA has been studied extensively and classified as oncomiR or tsmiR depending on the tumor type. For several cancer types, this miRNA has been identified to be downregulated in tumor tissues compared to normal tissues, for example, in lung cancer (53), breast cancer (54, 55), glioblastoma (56, 57), and hepatocellular carcinoma (58, 59). Many studies have identified KRAS as a direct target of miR-134-3p, thereby causing its tumor-suppressive effect (60). However, based on our microarray data, we did not observe downregulation of KRAS after miR-134-3p treatment. In line with our data, several studies have defined miR-134 as an oncomiR, for example, in lung cancer (49), uveal melanoma (50), prostate cancer (51), and colon cancer (52). In human melanoma patients high miR-134 expression level shows a tendency to be associated with worse prognosis (**Supplementary Figure 12**).

Our screen identified miR-224-3p as a miRNA that enhances NT5E expression, thus classifying miR-224-3p as an oncomiR. Indeed, several studies have assigned miR-224-3p oncogenic properties. For example, miR-224-3p levels elevated in NSCLC were found to increase cancer cell proliferation and inhibit apoptotic processes; sponging of this miRNA by lncRNA HCG11 could prevent the oncogenic functions of miR-224-3p in NSCLC cells (61). Furthermore, miR-224 levels were found to be upregulated in colorectal and bladder cancers, and miR-224 expression could drive tumor cell proliferation, migration, and invasion in colorectal cancer (62, 63). In addition, high miR-224-3p serum levels have been postulated as diagnostic markers for colon cancer (64). Based on the survival data of breast cancer patients, we found that high miR-224 expression was significantly associated with poor outcomes (**Supplementary Figure S11**).

Notably, miR-155-5p is one of the few miRNAs that decreased the expression of more than one ICM. Based on the library screen and subsequent validation experiments, we found that this miRNA lowered NT5E and CD274 surface expression (manuscript in preparation). Consistently, the regulation of CD274 expression by miR-155-5p has already been described in lung adenocarcinoma (65); however, the classification of miR-155-5p as an oncomiR or tsmiR remains controversial (66–72). Interestingly, when looking into human tumor patient expression data, we noticed a strong positive correlation between NT5E and MIR155HG mRNA expression level (**Supplementary Figure S13**). This was also observed for CD274 and MIR155HG mRNA levels. This indicates a negative feedback loop, since miR-155-5p was proven in our study to target and inhibit both CD274 and NT5E expression. On the other hand, high expression level of MIR155HG gene is significantly associated with better survival in human melanoma patients (**Supplementary Figure S13**) indicating its potential role as a tsmiRNA. Also, in breast cancer the same tendency can be observed. Furthermore, high expression level of the miRNA miR-155 itself is significantly linked to better prognosis of both melanoma and breast cancer patients.

## 5 Conclusion

In this study, miRNAs inhibiting ICM expression such as miR-1285-5p, miR-3134, and miR-155-5p were identified, which might be relevant in tumor immunotherapy approaches aimed at neutralizing the immunosuppressive tumor microenvironment. Moreover, we found miRNAs that boost ICM expression, and may therefore possibly be involved in the mechanisms of tumor immune escape. These potential oncomiRs may serve as therapeutic targets or prognostic markers in the clinic. A better understanding of the mechanisms underlying miRNA-mediated enhancement of ICM expression might uncover new therapeutic targets or pathways for cancer immunotherapy.

## Data availability statement

The original contributions presented in the study are included in the article/**Supplementary Material**, further inquiries can be directed to the corresponding author/s.

## Author contributions

Conception and design: SE. Development of methodology: TK. Acquisition of data (provided animals, acquired and managed patients, and provided facilities, etc.): TK and T-YC. Analysis and interpretation of data (e.g., statistical analysis, biostatistics, and computational analysis): TK, WO, and SE. Writing, review, and/or revision of the manuscript: TK, WO, and SE. Administrative, technical, or material support (i.e., reporting or organizing data, constructing databases): Not applicable. Study supervision: SE. Parts of the data used for this study have been acquired during the PhD thesis of the first author. All authors contributed to the article and approved the submitted version.
