# Supplementary material for: Novel microRNAs modulating ecto-5′-nucleotidase expression
Source: Front Immunol. 2023 Jun 20;14:1199374. doi: 10.3389/fimmu.2023.1199374 (PMC10318900; doi:10.3389/fimmu.2023.1199374)
Supplement: Supplementary file 1 [file DataSheet_1.pdf]

frontiers

*Supplementary Material*

# Novel microRNAs modulating ecto-5′-nucleotidase expression

**Theresa Kordaß[1,2], Tsu-Yang Chao[1], Wolfram Osen[1], Stefan B. Eichmüller[1*]**

[1]GMP & T Cell Therapy Unit, German Cancer Research Center (DKFZ), Heidelberg, Germany

[2]Faculty of Biosciences, University Heidelberg, Germany

*** Correspondence:**
S. B. Eichmüller
s.eichmueller@dkfz.de

## 1 Supplementary Methods

### 1.1 *In silico* miRNA prediction

Ten publicly available data bases were used to determine miRNAs predicted to regulate CD274, CTLA4, ENTPD1 and NT5E. The following resources were downloaded at 01-05-2017 and used for *in silico* predictions:

- MicroCosm [1]
- miRanda: microRNA conserved and non-conserved [2]
- miRDB V5 [3]
- miRecords [4]
- miRGator [5]
- miRNA Map [6]
- PACCMIT [7]
- PicTar [8]
- PITA Top Score and all predictions [9]
- TargetScan [10]

### 1.2 miRNA transfection

Results of the miRNA library screen were validated by individual transfections as follows: 2 x $10^5$ cells were seeded in 12 well plate and cultured for 24 h reaching approx. 70 % confluency. Cells were transfected with 50 nM miRNA or siRNA using Lipofectamine RNAiMax reagent according to the manufacturer’s protocol. Cells were harvested for RNA isolation and subsequent qPCR or Microarray analysis 48 h post transfection. Expression of cell surface molecules was measured by FACS 72 h post transfection.

### 1.3 RNA extraction and qPCR

Total RNA from frozen cell pellets was isolated using miRNeasy Mini Kit according to the manufacturer's protocol, enabling simultaneous extraction of miRNA and mRNA for expression analysis. Cell pellets were lysed using with phenol/guanidine based QIAzol lysis reagent. Subsequently, RNA was purified with silica-membrane spin columns and eluted with 30 µL nuclease-free water. In case of mRNA expression analysis, total RNA was isolated using the RNAeasy Kit according to the manufacturer's protocol. RNA samples were stored at -80 °C until further use for cDNA synthesis. RNA concentrations were measured with a NanoDrop spectrophotometer (ThermoFisher Scientific, Waltham, USA) or Qubit device using the RNA BR Assay kit (ThermoFisher Scientific, Waltham, USA). For mRNA expression analysis, 500 ng of total RNA were used for reverse transcription in a 20 µL reaction volume with oligo(dT)18 primers applying the Transcriptor First Strand cDNA Synthesis Kit (Roche Applied Science, Mannheim, Germany). To analyze miRNA expression, 40 ng of total RNA were reverse transcribed in a 15 µL reaction volume using TaqMan™ MicroRNA reverse transcription kit (Applied Biosystems, Foster City, CA, USA) according to the manufacturer's protocol including specific stem-loop primers for mature miRNA. All PCRs were run on a Veriti 96 well Thermal Cycler or QuantStudio 3 Real-Time PCR System (ThermoFisher Scientific, Waltham, USA). To perform qPCR with cDNA synthesized from mRNA, 2 µL cDNA diluted 1:5 with PCR-quality water was used in a 20 µL reaction volume using the SYBR Green PowerUP PCR Mastermix for qPCR. RPL19 and TBP encoding genes lacking putative binding site for transfected miRNAs were included as house-keeping genes for normalization. For cDNA synthesis from miRNA, 2 µL miRNA were used in a per PCR reaction performed in a total volume of 20 µL. Small nuclear RNA U6 (RNU6B) was used as endogenous control. For all samples three technical replicates were performed and relative expression was calculated according to the $2^{-\Delta\Delta Ct}$-method.

## 2 Supplementary Figures and Tables

### 2.1 Supplementary Tables

**Table S1.** Antibodies used for flow cytometry.

| Antibody | Type | Catalogue # | Manufacturer |
|---|---|---|---|
| **PE anti-human CD73** | IgG1, κ | 344003 | Biolegend, San Diego, USA |
| **BB515 anti-human CD39** | IgG2b, κ | 565469 | Becton Dickinson, Franklin Lakes, USA |
| **PE-Cy7 anti-human CD274** | IgG1, κ | 25-5983-41 | Invitrogen, Carlsbad, USA |
| **PE isotype control** | IgG1, κ | 12-4714-81 | eBioscience, San Diego, USA |
| **PE-Cy7 isotype control** | IgG1, κ | 400125 | eBioscience, San Diego, USA |
| **BB515 isotype control** | IgG2b, κ | 564510 | Becton Dickinson, Franklin Lakes, USA |

**Table S2.** List of miRNAs used in this study.

| miRNA | Sequence 5'-3' | Purpose |
|---|---|---|
| **ath-miR-416** | GGUUCGUACGUACACUGUUCA | Negative miRNA control 1 |
| **cel-miR-243-3p** | CGGUACGAUCGCGGCGGGAUAUC | Negative miRNA control 2 |
| **hsa-miR-127-5p** | CUGAAGCUCAGAGGGCUCUGAU | Screen hit |
| **hsa-miR-1233-3p** | UGAGCCCUGUCCUCCCGCAG | Screen hit |
| **hsa-miR-1293** | UGGGUGGUCUGGAGAUUUGUGC | Screen hit |
| **hsa-miR-1298-3p** | CAUCUGGGCAACUGACUGAAC | Screen hit |
| **hsa-miR-134-3p** | CCUGUGGGCCACCUAGUCACCAA | Screen hit |
| **hsa-miR-143-5p** | GGUGCAGUGCUGCAUCUCUGGU | Screen hit |
| **hsa-miR-148b-3p** | UCAGUGCAUCACAGAACUUUGU | Screen hit |
| **hsa-miR-155-5p** | UUAAUGCUAAUCGUGAUAGGGGUU | Screen hit |
| **hsa-miR-16-5p** | UAGCAGCACGUAAAUAUUGGCG | Screen hit |
| **hsa-miR-181b-2-3p** | CUCACUGAUCAAUGAAUGCA | Screen hit |
| **hsa-miR-193a-3p** | UGGGUCUUUGCGGGCGAGAUGA | Screen hit |
| **hsa-miR-193b-3p** | AACUGGCCCUCAAAGUCCCGCU | Screen hit |
| **hsa-miR-22-3p** | AAGCUGCCAGUUGAAGAACUGU | Screen hit |
| **hsa-miR-224-3p** | AAAAUGGUGCCCUAGUGACUACA | Screen hit |
| **hsa-miR-3116** | UGCCUGGAACAUAGUAGGGACU | Screen hit |
| **hsa-miR-3118** | UGUGACUGCAUUAUGAAAAUUCU | Screen hit |
| **hsa-miR-3126-5p** | UGAGGGACAGAUGCCAGAAGCA | Screen hit |
| **hsa-miR-3134** | UGAUGGAUAAAAGACUACAUAUU | Screen hit |
| **hsa-miR-3190-5p** | UCUGGCCAGCUACGUCCCCA | Screen hit |
| **hsa-miR-422a** | ACUGGACUUAGGGUCAGAAGGC | Published miRNA |
| **hsa-miR-4480** | AGCCAAGUGGAAGUUACUUUA | Screen hit |
| **hsa-miR-4672** | UUACACAGCUGGACAGAGGCA | Screen hit |
| **hsa-miR-4692** | UCAGGCAGUGUGGGUAUCAGAU | Screen hit |
| **hsa-miR-520d-3p** | AAAGUGCUUCUCUUUGGUGGGU | Screen hit |
| **hsa-miR-6514-3p** | CUGCCUGUUCUUCCACUCCAG | Screen hit |
| **hsa-miR-6859-3p** | UGACCCCCAUGUCGCCUCUGUAG | Screen hit |

**Table S3.** Composition of phosphate-free buffers.

| Chemical | Stock | MW [g/mol] | Preparation stock | Vol. for 450 mL | Final concentration |
|---|---|---|---|---|---|
| **$MgCl_2$** | 2 M | 203.30 | 0.82 g in 2 mL | 450 µl | 2 mM |
| **NaCl** | 1.25 M | 58.44 | 3.65 g in 50 mL | 45 mL | 125 mM |
| **KCl** | 1 M | 74.56 | 3.70 g in 50 mL | 450 µL | 1 mM |
| **Glucose** | 138 mM | 198.17 | 1.40 g in 50 mL | 32.4 mL | 10 mM |
| **HEPES** | 2 M | 238.31 | 2.40 g in 10 mL | 4.5 mL | 10 mM |
| **$ddH_20$** | | | | 367.2 mL | |

**Table S4.** List of miRNAs predicted by at least 3 databases to regulate NT5E expression. No. DB, number of databases.

| miRNA | No. DB | miRNA | No. DB |
|---|---|---|---|
| **miR-422a** | 6 | **miR-513** | 3 |
| **miR-518a-5p** | 5 | **miR-580** | 3 |
| **miR-548l** | 5 | **miR-587** | 3 |
| **miR-22** | 4 | **miR-630** | 3 |
| **miR-507** | 4 | **miR-650** | 3 |
| **miR-557** | 4 | **miR-662** | 3 |
| **miR-140-5p** | 4 | **miR-654** | 3 |
| **miR-590-3p** | 4 | **miR-657** | 3 |
| **miR-527** | 4 | **miR-129** | 3 |
| **miR-1246** | 4 | **miR-125** | 3 |
| **miR-448** | 3 | **miR-548** | 3 |
| **miR-524-5p** | 3 | **miR-182** | 3 |
| **miR-363** | 3 | **miR-378** | 3 |
| **miR-30d** | 3 | **miR-313** | 3 |
| **miR-30b** | 3 | **miR-316** | 3 |
| **miR-153** | 3 | **miR-318** | 3 |
| **miR-125a-5p** | 3 | **miR-320** | 3 |
| **miR-193a-3p** | 3 | **miR-378** | 3 |
| **miR-433** | 3 | **miR-431** | 3 |
| **miR-146b-5p** | 3 | **miR-428** | 3 |
| **miR-142-5p** | 3 | **miR-30a** | 3 |
| **miR-520h** | 3 | **miR-30e** | 3 |

**Table S5.** miRNAs predicted by at least 3 data bases to regulate CD274 expression.

| miRNA | No. DB | miRNA | No. DB |
|---|---|---|---|
| **miR-140-3p** | 4 | **miR-1205** | 3 |
| **miR-28-3p** | 4 | **miR-548e** | 3 |
| **miR-384** | 4 | **miR-548l** | 3 |
| **miR-140-5p** | 3 | **miR-1243** | 3 |
| **miR-429** | 3 | **miR-1270** | 3 |
| **miR-501-5p** | 3 | **miR-302f** | 3 |
| **miR-383** | 3 | **miR-513b** | 3 |
| **miR-422a** | 3 | **miR-1273c** | 3 |
| **miR-188-3p** | 3 | **miR-378c** | 3 |
| **miR-219-2-3p** | 3 | **miR-4264** | 3 |
| **miR-485-3p** | 3 | **miR-4279** | 3 |
| **miR-520h** | 3 | **miR-17-5p** | 3 |
| **miR-513a-5p** | 3 | **miR-320a** | 3 |
| **miR-563** | 3 | **miR-377-3p** | 3 |
| **miR-576-3p** | 3 | **miR-548a-5p** | 3 |
| **miR-548a-3p** | 3 | **miR-548b-5p** | 3 |
| **miR-636** | 3 | **miR-548i** | 3 |
| **miR-128** | 3 | **miR-558** | 3 |
| **miR-802** | 3 | **miR-93-5p** | 3 |
| **miR-1225-3p** | 3 | | |

**Table S6.** miRNAs predicted by at least three data bases to regulate CTLA4 expression.

| miRNA | No. DB | miRNA | No. DB |
|---|---|---|---|
| **miR-516a-3p** | 5 | **miR-579** | 3 |
| **miR-656** | 4 | **miR-581** | 3 |
| **miR-429** | 4 | **miR-582-3p** | 3 |
| **miR-324-5p** | 4 | **miR-583** | 3 |
| **miR-384** | 3 | **miR-548c-3p** | 3 |
| **miR-380** | 3 | **miR-1200** | 3 |
| **miR-155** | 3 | **miR-1287** | 3 |
| **miR-494** | 3 | **miR-1243** | 3 |
| **miR-496** | 3 | **miR-1248** | 3 |
| **miR-542-3p** | 3 | **miR-1261** | 3 |
| **miR-1297** | 3 | **miR-302f** | 3 |
| **miR-140-3p** | 3 | **miR-548p** | 3 |
| **miR-127-5p** | 3 | **miR-1279** | 3 |
| **miR-330-3p** | 3 | **miR-3182** | 3 |
| **miR-511** | 3 | **miR-4311** | 3 |
| **miR-502-5p** | 3 | **miR-4282** | 3 |
| **miR-502-3p** | 3 | | |

**Table S7.** miRNAs predicted by at least 2 data bases to regulate ENTPD1 expression.

| miRNA | No. DB | miRNA | No. DB |
|---|---|---|---|
| **miR-421** | 3 | **miR-30c** | 2 |
| **miR-140-5p** | 3 | **miR-30b** | 2 |
| **miR-346** | 3 | **miR-140-3p** | 2 |
| **miR-300** | 2 | **miR-573** | 2 |
| **miR-146b-5p** | 2 | **miR-595** | 2 |
| **miR-381** | 2 | **miR-607** | 2 |
| **miR-144** | 2 | **miR-649** | 2 |
| **miR-337-5p** | 2 | **miR-802** | 2 |
| **miR-520e** | 2 | **miR-1276** | 2 |
| **miR-193a-3p** | 2 | **miR-3175** | 2 |
| **miR-338-3p** | 2 | **miR-15a-5p** | 2 |
| **miR-766** | 2 | **miR-15b-5p** | 2 |
| **miR-526b** | 2 | **miR-16-5p** | 2 |
| **miR-936** | 2 | **miR-195-5p** | 2 |
| **miR-520a-5p** | 2 | **miR-2110** | 2 |
| **miR-499-5p** | 2 | **miR-424-5p** | 2 |
| **miR-328** | 2 | **miR-429** | 2 |
| **let-7e** | 2 | **miR-4428** | 2 |
| **miR-520b** | 2 | **miR-4742-3p** | 2 |
| **miR-625** | 2 | **miR-497-5p** | 2 |
| **miR-545*** | 2 | **miR-548l** | 2 |
| **miR-23b** | 2 | **miR-641** | 2 |
| **miR-708** | 2 | **miR-590-3p** | 2 |
| **miR-16-2*** | 2 | **miR-331-3p** | 2 |
| **miR-630** | 2 | **miR-330-5p** | 2 |
| **miR-27b*** | 2 | **miR-136** | 2 |
| **miR-920** | 2 | **miR-326** | 2 |
| **miR-493** | 2 | **miR-194** | 2 |
| **miR-545** | 2 | **miR-205** | 2 |
| **miR-30b*** | 2 | | |

**Table S8.** miRNAs predicted to target the entire panel of immune checkpoint molecules investigated (CD274, CTLA4, NT5E and ENTPD1). miRNAs predicted by at least one of the data bases employed to target all four immune checkpoint molecules were considered.

| miRNA | miRNA |
|---|---|
| miR-422a | miR-378 |
| miR-548l | miR-374b |
| miR-590-3p | miR-582-5p |
| miR-1246 | miR-584 |
| miR-662 | miR-1208 |
| miR-656 | miR-548m |
| miR-338-5p | miR-501-3p |
| miR-548n | miR-193b |
| miR-28-5p | miR-335* |
| miR-495 | miR-548k |
| miR-330-3p | miR-326 |
| miR-511 | miR-142-3p |
| miR-3148 | miR-519d |
| miR-496 | miR-568 |
| miR-485-3p | miR-548f |
| miR-641 | miR-1206 |
| miR-607 | miR-29b-2* |
| miR-490-5p | miR-512-3p |
| miR-410 | miR-340-5p |
| miR-875-5p | miR-138 |
| miR-708 | miR-150 |
| miR-491-3p | miR-193a-5p |
| miR-219-2-3p | |
| miR-155 | |
| miR-487a | |
| miR-1248 | |
| miR-548a-3p | |

**Table S9.** miRNA top hits affecting NT5E surface expression in both cell lines, MDA-MB-231 (MDA) and SK-Mel-28 (SK28). miRNA showing significant effects only in one of the two cell lines are marked by asterisk. For each miRNA the deep sequencing data/reads (obtained from miRBase) were added to preferentially select already confirmed miRNAs, which we define as miRNAs with read > 40. miRNAs marked in red have read < 40 and were excluded for further validation.

| **miRNAs enhancing NT5E expression** | | | | | |
|---|---|---|---|---|---|
| **miRNA** | **BS** | **z-score MDA** | **z-score SK28** | **sum** | **reads** |
| **miR-134-3p** | 0 | 1.99 | 4.87 | 6.86 | **199** |
| **miR-6514-3p** | 0 | 2.62 | 2.91 | 5.53 | **786** |
| **miR-593-5p** | 1 | 2.21 | 2.11 | 4.32 | **1** |
| **miR-6859-3p** | 0 | 2.5 | 1.66 | 4.16 | **1388** |
| **miR-3126-5p** | 1 | 2.07 | 1.85 | 3.92 | **241** |
| **miR-4672** | 1 | 2.23 | 1.89 | 3.89 | **44** |
| **miR-4692** | 1 | 1.79 | 2.04 | 3.83 | **6** |
| **miR-16-5p** | 0 | 2.1 | 1.69 | 3.79 | **7830515** |
| **miR-3116*** | 0 | 2.5 | 0.98 | 3.48 | **303** |
| **miR-127-5p** | 1 | 1.73 | 1.67 | 3.4 | **207374** |
| **miR-224-3p** | 1 | 1.65 | 1.74 | 3.39 | **5757** |
| **miR-34b-3p*** | 0 | 1.39 | 2 | 3.39 | **3180** |
| **miR-1293*** | 0 | 1.76 | 1.35 | 3.11 | **173** |
| **miRNAs inhibiting NT5E expression** | | | | | |
| **miRNA** | **BS** | **z-score MDA** | **z-score SK28** | **sum** | **reads** |
| **miR-1285-5p** | 2 | -2.27 | -2.55 | -4.82 | **17819** |
| **miR-193a-3p** | 1 | -2.48 | -2.32 | -4.80 | **72316** |
| **miR-181b-2-3p** | 0 | -2.01 | -2.56 | -4.57 | **29357** |
| **miR-22-3p** | 1 | -1.72 | -2.78 | -4.50 | **1254963** |
| **miR-3134** | 2 | -2.27 | -2.19 | -4.46 | **132** |
| **miR-5584-3p** | 2 | -1.82 | -2.22 | -4.04 | **5** |
| **miR-5190** | 1 | -2.27 | -1.70 | -3.97 | **31** |
| **miR-4480** | 1 | -2.20 | -1.72 | -3.92 | **5** |
| **miR-143-5p** | 1 | -2.09 | -1.70 | -3.79 | **458908** |
| **miR-1298-3p** | 1 | -1.86 | -1.91 | -3.77 | **487** |
| **miR-3118** | 2 | -1.70 | -2.03 | -3.73 | **344** |
| **miR-4676-5p** | 1 | -1.80 | -1.83 | -3.63 | **40** |
| **miR-148b-3p** | 1 | -1.80 | -1.78 | -3.58 | **1313333** |
| **miR-155-5p** | 2 | -1.78 | -1.76 | -3.54 | **177692** |
| **miR-8056** | 2 | -1.76 | -1.71 | -3.47 | **3** |
| **miR-520d-3p** | 1 | -1.66 | -1.76 | -3.42 | **58** |
| **miR-193b-3p*** | 1 | -1.83 | -0.50 | -2.33 | **163980** |

**Table S10.** List of constructs used in reporter assays to proof direct binding of miRNAs to the NT5E 3'-UTR. The forward and reverse primers for site-specific quick-change mutagenesis are given for each construct. Primer sequences were designed with QuikChange Primer Design tool (https://www.agilent.com/store/primerDesignProgram.jsp).

| Construct | Primer | Purpose |
|---|---|---|
| **del-1670** | fwd: ATTTAGGGTTTATTTTTTACACTTGGCAGTAAAATAGGGTAAATCCTATTAG<br>rev: CTAATAGGATTTACCCTATTTTACTGCCAAGTGTAAAAAATAAACCCTAAAT | mutation BS<br>miR-193a-3p |
| **del-1442** | fwd: CTTAAAAACAGTGTGCAAATGGAGCTAGAGGTTTTGATAGGAAG<br>rev: CTTCCTATCAAAACCTCTAGCTCCATTTGCACACTGTTTTTAAG | mutation BS<br>miR-22-3p |
| **del-1443** | fwd: AAACAGTGTGCAAATGGCGCTAGAGGTTTTGATAGG<br>rev: CCTATCAAAACCTCTAGCGCCATTTGCACACTGTTT | mutation BS<br>miR-22-3p |
| **del-1444** | fwd: AAAACAGTGTGCAAATGGCACTAGAGGTTTTGATAGGAAG<br>rev: CTTCCTATCAAAACCTCTAGTGCCATTTGCACACTGTTTT | mutation BS<br>miR-22-3p |
| **del-88** | fwd: CTAAAAGGCAGATTTGAATCTACTTGAAAAAAATGCAGTTTCACACATTA<br>rev: TAATGTGTGAAACTGCATTTTTTTCAAGTAGATTCAAATCTGCCTTTTAG | mutation BS<br>miR-1285-5p |
| **del-89** | fwd: GTCCTAAAAGGCAGATTTGAATCCACTTGAAAAAAATGCAGTTTCAC<br>rev: GTGAAACTGCATTTTTTTCAAGTGGATTCAAATCTGCCTTTTAGGAC | mutation BS<br>miR-1285-5p |
| **del-90** | fwd: TGAAACTGCATTTTTTTCAAGTGAATTCAAATCTGCCTTTTAGGACC<br>rev: GGTCCTAAAAGGCAGATTTGAATTCACTTGAAAAAAATGCAGTTTCA | mutation BS<br>miR-1285-5p |
| **del-884** | fwd: AGGCAGAGCTGATGGAATCCATAAAATAACAGCTAATGC<br>rev: GCATTAGCTGTTATTTTATGGATTCCATCAGCTCTGCCT | mutation BS<br>miR-1285-5p |
| **del-885** | fwd: GGCAGAGCTGATGGAATTCATAAAATAACAGCTAATGCC<br>rev: GGCATTAGCTGTTATTTTATGAATTCCATCAGCTCTGCC | mutation BS<br>miR-1285-5p |
| **del-886** | fwd: GAGGCAGAGCTGATGGAACTCATAAAATAACAGCTAATGC<br>rev: GCATTAGCTGTTATTTTATGAGTTCCATCAGCTCTGCCTC | mutation BS<br>miR-1285-5p |
| **del-352** | fwd: CATATTTTTCTTCTTCATATCCATTTCTAATCATCAAACAGCTTATGTTTACATAAAATTT<br>rev: AAATTTTATGTAAACATAAGCTGTTTGATGATTAGAAATGGATATGAAGAAGAAAAATATG | mutation BS<br>miR-3134 |
| **del-353** | fwd: CATATTTTTCTTCTTCATATCCATTTCTAATCATCAAACAGCTTATGTTTACATAAAATTTTAT<br>rev: ATAAAATTTTATGTAAACATAAGCTGTTTGATGATTAGAAATGGATATGAAGAAGAAAAATATG | mutation BS<br>miR-3134 |
| **del-354** | fwd: TTTTTCTTCTTCATATCCATTTCTAATCCTCAAACAGCTTATGTTTACATAAAATTTT<br>rev: AAAATTTTATGTAAACATAAGCTGTTTGAGGATTAGAAATGGATATGAAGAAGAAAAA | mutation BS<br>miR-3134 |
| **del-889** | fwd: GAGGCAGAGCTGATGAATCTCATAAAATAACAGCTAATGCCG<br>rev: CGGCATTAGCTGTTATTTTATGAGATTCATCAGCTCTGCCTC | mutation BS<br>miR-3134 |
| **del-990** | fwd: GGACAGAGGCAGAGCTGATGAATCTCATAAAATAACAGCT<br>rev: AGCTGTTATTTTATGAGATTCATCAGCTCTGCCTCTGTCC | mutation BS<br>miR-3134 |
| **del-991** | fwd: GTTATTTTATGAGATTCCTCAGCTCTGCCTCTGTCC<br>rev: GGACAGAGGCAGAGCTGAGGAATCTCATAAAATAAC | mutation BS<br>miR-3134 |

**Table S11.** Microarray analysis after miRNA transfection performed on SK-Mel28, MDA-MB-231 and MaMel-02 cells 48 h after treatment. The miRNAs tested in the respective cell lines are listed.

| SK-Mel-28 | MaMel-02 | MDA-MB-231 |
|---|---|---|
| mimic control-1 | mimic control-1 | mimic control-1 |
| miR-134-3p | miR-134-3p | miR-134-3p |
| miR-3126-5p | miR-3126-5p | miR-3126-5p |
| miR-34b-3p | miR-34b-3p | miR-34b-3p |
| miR-4672 | miR-4672 | miR-4672 |
| miR-6859-3p | miR-6859-3p | miR-6859-3p |
| miR-1293 | miR-1293 | |
| miR-3116 | miR-3116 | |
| miR-224-3p | | miR-224-3p |
| miR-6514-5p | | miR-6514-5p |
| miR-127-5p | | |

**Table S12.** For NT5E enhancing miRNAs the number of potential binding sites with 3'-UTR of selected target genes was analyzed by miRMap tool.

| miRNA | CBX6 | CNOT6L | NFATC3 | SRSF4 |
|---|---|---|---|---|
| **miR-16-5p** | 5 | 6 | 4 | 0 |
| **miR-127-5p** | 2 | 1 | 0 | 0 |
| **miR-1293** | 8 | 0 | 1 | 0 |
| **miR-134-3p** | 7 | 2 | 1 | 3 |
| **miR-224-3p** | 1 | 8 | 1 | 2 |
| **miR-3116** | 11 | 0 | 1 | 1 |
| **miR-3126-5p** | 6 | 1 | 1 | 0 |
| **miR-34b-3p** | 4 | 1 | 1 | 2 |
| **miR-4672** | 0 | 6 | 0 | 0 |
| **miR-6514-3p** | 6 | 1 | 0 | 0 |
| **miR-6859-3p** | 5 | 0 | 0 | 0 |
| | **10/11** | **8/11** | **7/11** | **4/11** |

## 2.2 Supplementary Figures

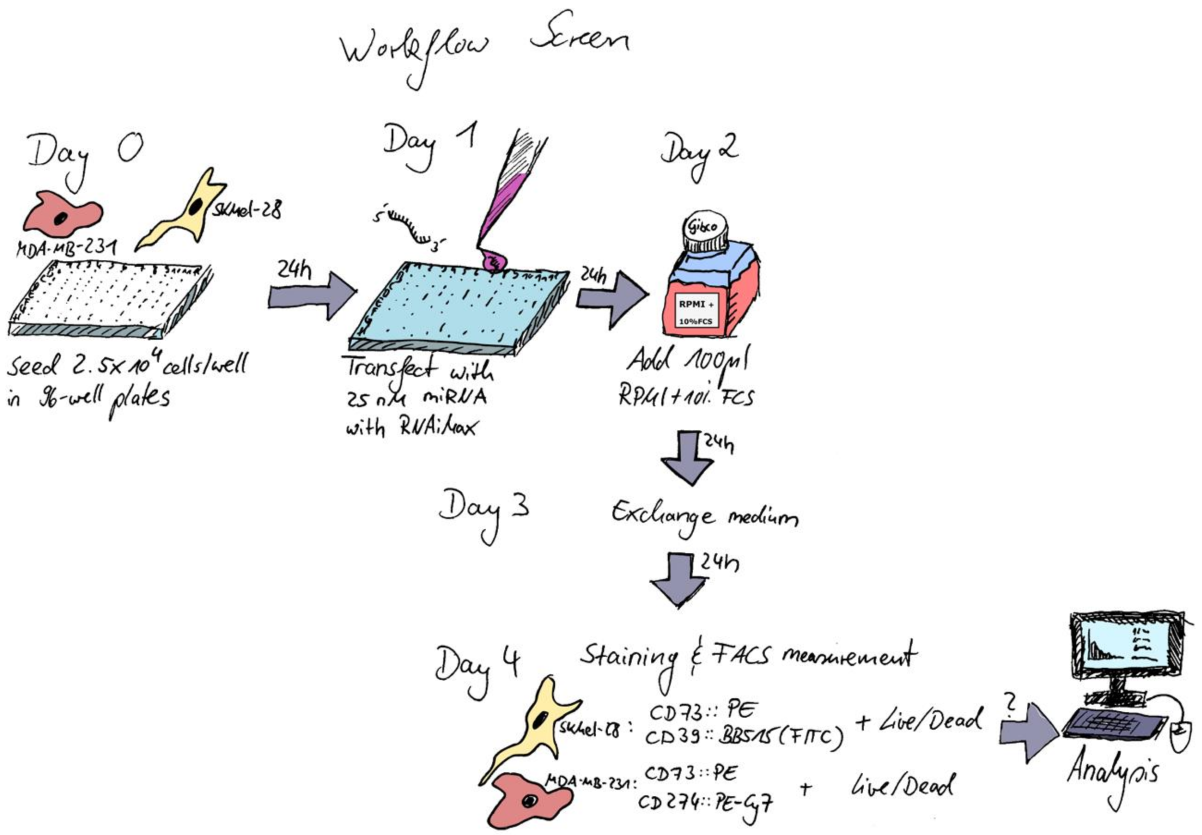


**Supplementary Figure 1. Workflow of the miRNA library screen.** To identify miRNAs capable of modulating surface expression of selected immune checkpoint molecules on human cancer cell lines MDA-MB-231 (breast cancer) and SK-Mel-28 (melanoma) a miRNA library screen was conducted. Therefore, cells were seeded in 96-well plates and transfected with 25 nM miRNA using RNAiMax reagent. On day four cells were stained for flow cytometry measurement. MDA-MB-231 cells were stained CD73/NT5E and CD274/PD-L1 and SK-Mel-28 cells were stained for CD73/NT5E and CD39/ENTPD1. Both cell lines were additionally stained with Pacific orange to discriminate live and dead cells. FlowJo software was used to analyze the flow cytometry data. Median Fluorescence intensity values of the immune checkpoint molecules were exported and data analysis was conducted with R.

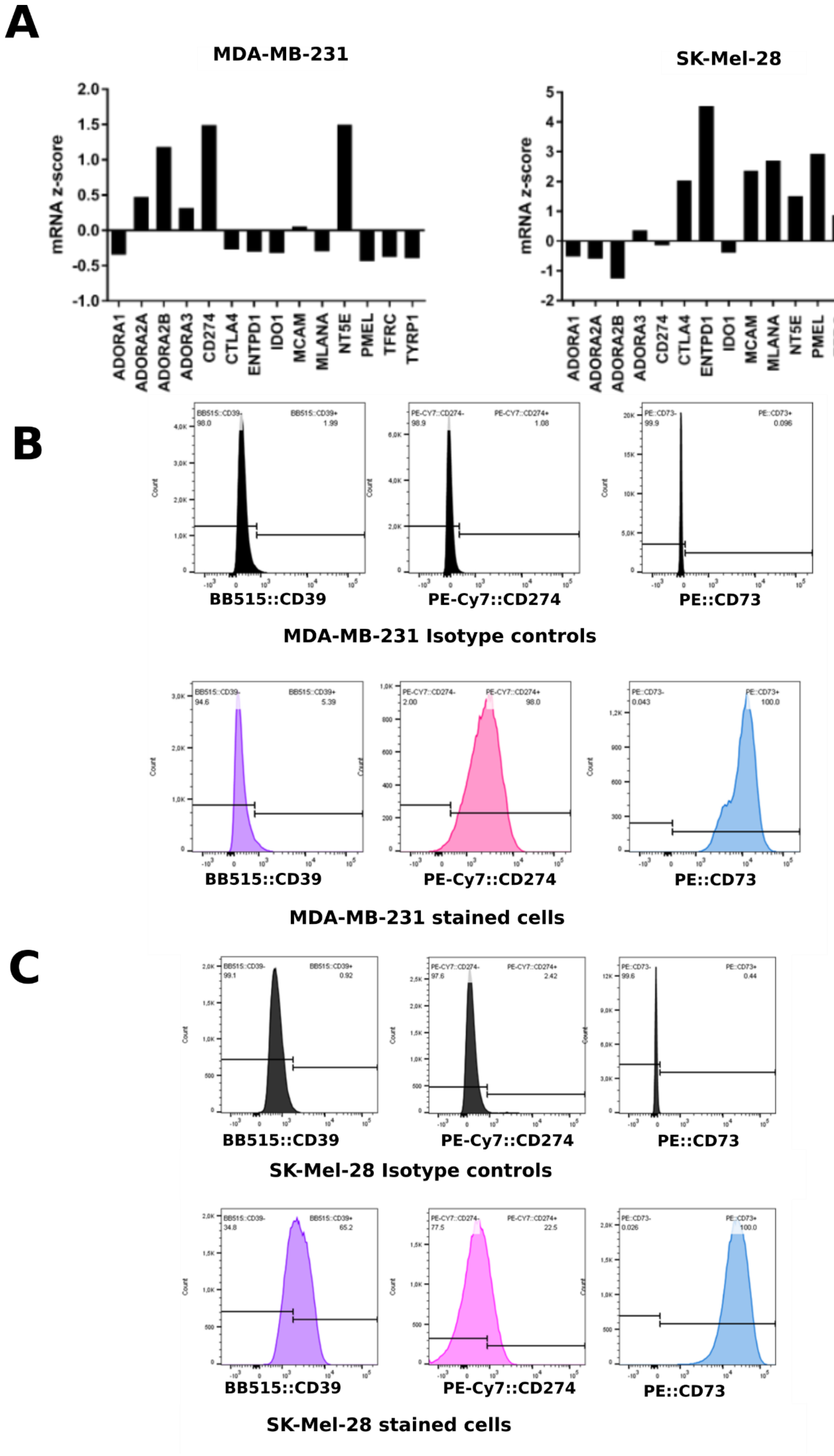
A
MDA-MB-231
SK-Mel-28
mRNA z-score
ADORA1
ADORA2A
ADORA2B
ADORA3
CD274
CTLA4
ENTPD1
IDO1
MCAM
MLANA
NT5E
PMEL
TFRC
TYRP1
B
BB515::CD39
PE-Cy7::CD274
PE::CD73
MDA-MB-231 Isotype controls
MDA-MB-231 stained cells
C
SK-Mel-28 Isotype controls
SK-Mel-28 stained cells

**Supplementary Figure 2. Expression level of selected genes related to CTL mediated tumor cell lysis.** (A) Expression levels of the selected genes in MDA-MB-231 and SK-Mel-28 cell lines were obtained from NCI-60 data set. mRNA levels are z-score normalized. Level of CD274, CD39 and CD73 surface expression was measured by flow cytometry for (B) MDA-MB-231 cells and (C) SK-Mel-28 cells. Both cell lines express high levels of NT5E. MDA-MB-231 also exhibits high expression levels of the immune checkpoint molecule CD274. SK-Mel-28 also shows high levels of melanoma-associated antigens including MLANA. Furthermore, SK-Mel-28 is one of the few cell lines within the NCI-60 panel with high ENTPD1 expression. Besides NT5E, no immune checkpoint molecule was shared by both cell lines. Thus, for the miRNA library screen NT5E and ENTPD1 were selected for SK-Mel-28 and NT5E and CD274 for MDA-MB-231, respectively.

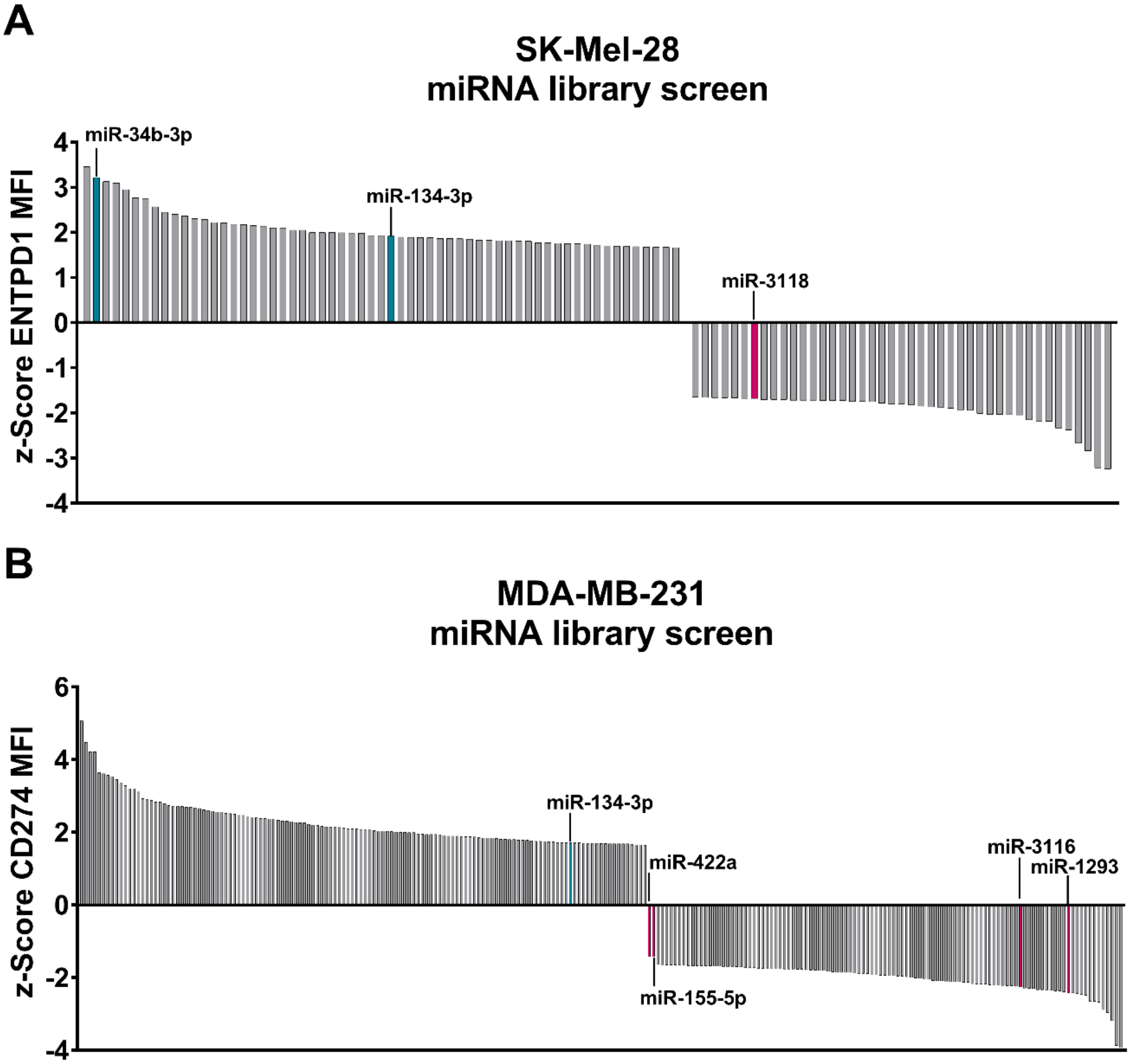


**Supplementary Figure 3. A comprehensive miRNA library screen reveals miRNAs affecting CD274/ENTPD1 surface expression in human tumor cell lines.** The human breast cancer cell line SK-Mel-28 **(A)** and human melanoma cell line MDA-MB-231 **(B)** were transfected with human miRNA library and changes in ENTPD1 surface expression (A) or CD274 surface expression (B) was measured by flow cytometry 72 h post transfection. The median fluorescence intensity values (MFI) were z-score normalized for each plate. Modulating miRNAs selected for further validation are depicted in turquois (enhancing immune checkpoint expression) and magenta (decreasing immune checkpoint expression), respectively.

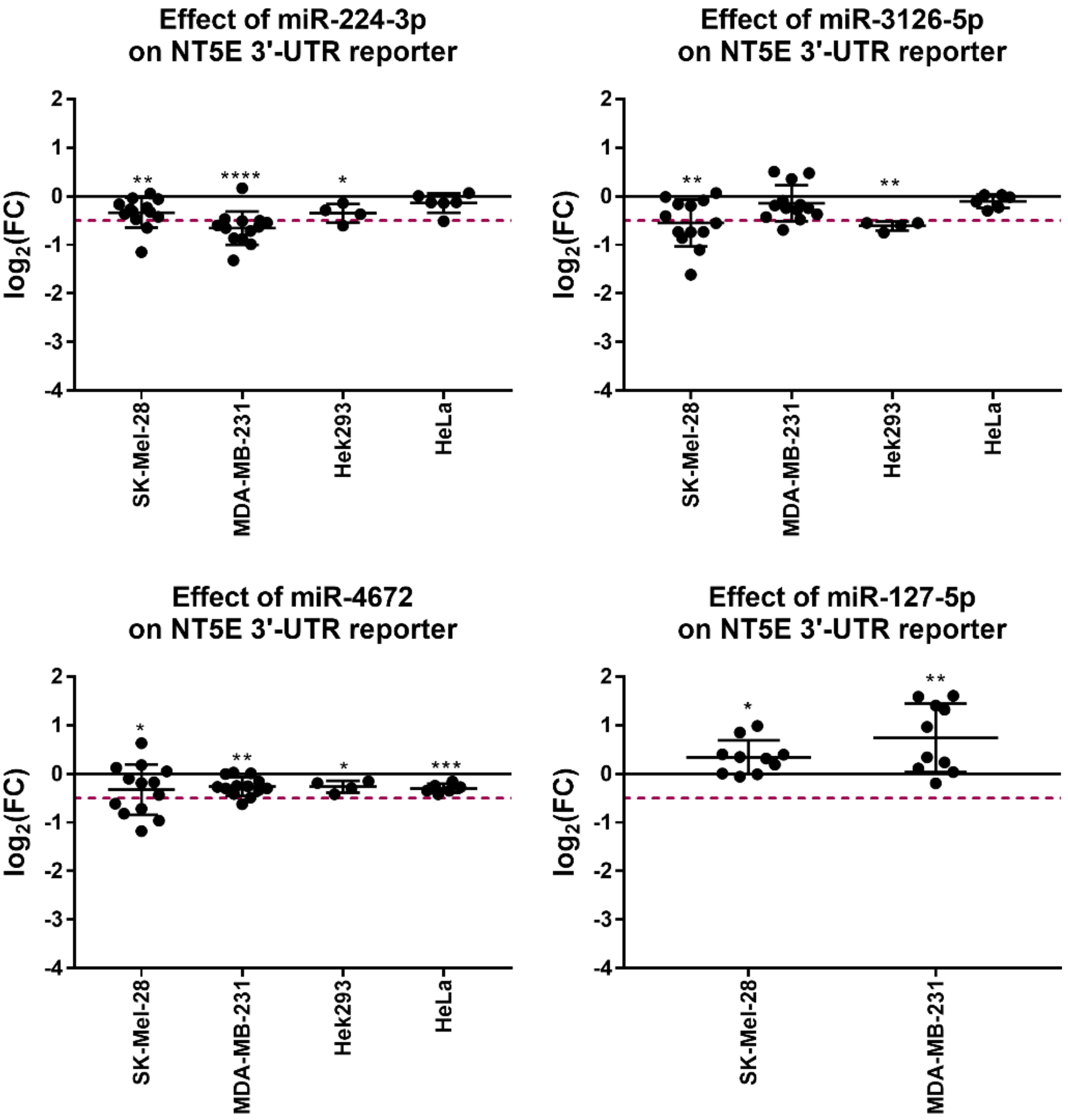


**Supplementary Figure 4: NT5E-3'-UTR luciferase reporter assay performed with NT5E enhancing miRNAs**. miRNAs with enhancing effects on NT5E surface expression containing at least one predicted binding for the NT5E 3'-UTR were tested in NT5E reporter assays. Cells were transfected with 25 nM miRNAs in 96-well format. 24 h post transfection, luciferase activity was measured. Fold changes in luminescence signal intensity were calculated compared to respective mimic control-1 samples. Significance was assessed by one-sample T-test. Luminesce signal was normalized to level of control transfections. Mean ± SD are shown. *: $p < 0.05$; **: $p < 0.01$; ***: $p < 0.001$; ****: $p < 0.0001$.

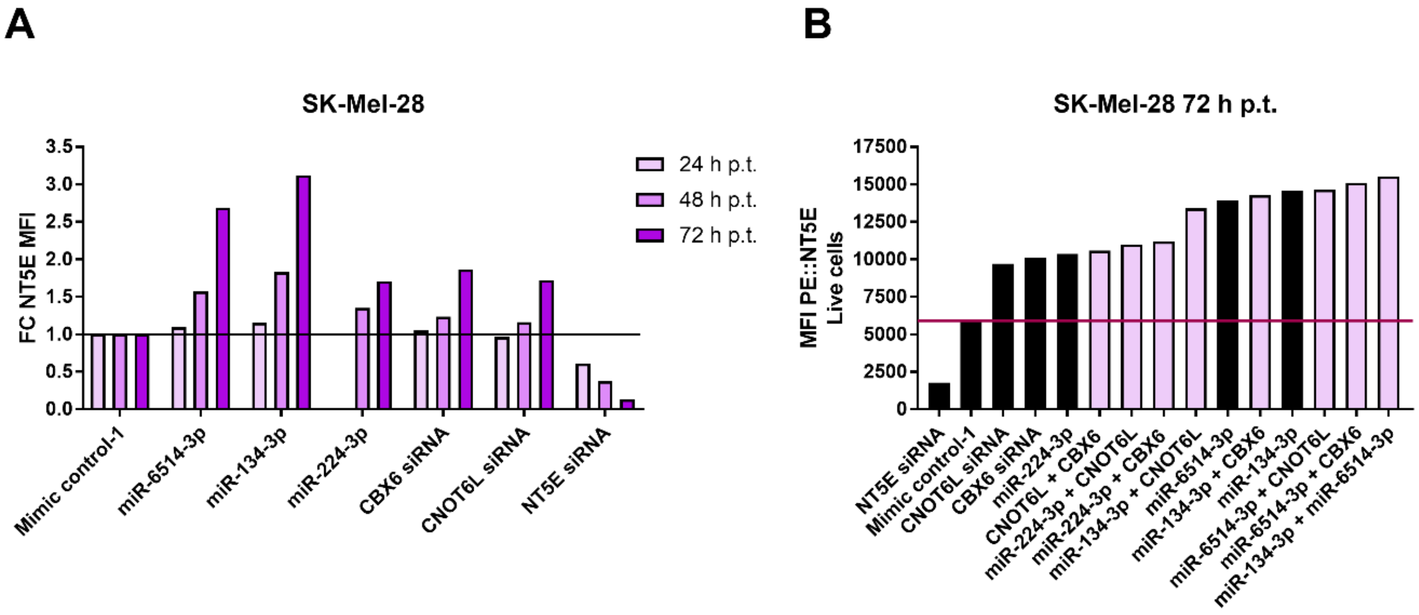


**Supplementary Figure 5: Comparing siRNA and miRNA mediated up-regulation of NT5E surface levels**. **(A)** SK-Mel-28 cells were transfected with 50 nM miRNA or siRNA pools and NT5E surface levels were measured by flow cytometry 24 h, 48 h and 72 h after treatment of the cells. **(B)** SK-Mel-28 cells were transfected with total amount of 50 nM miRNA/siRNA and NT5E surface levels were measured 72 h after transfection. Combination treatments are highlighted in pale violet.

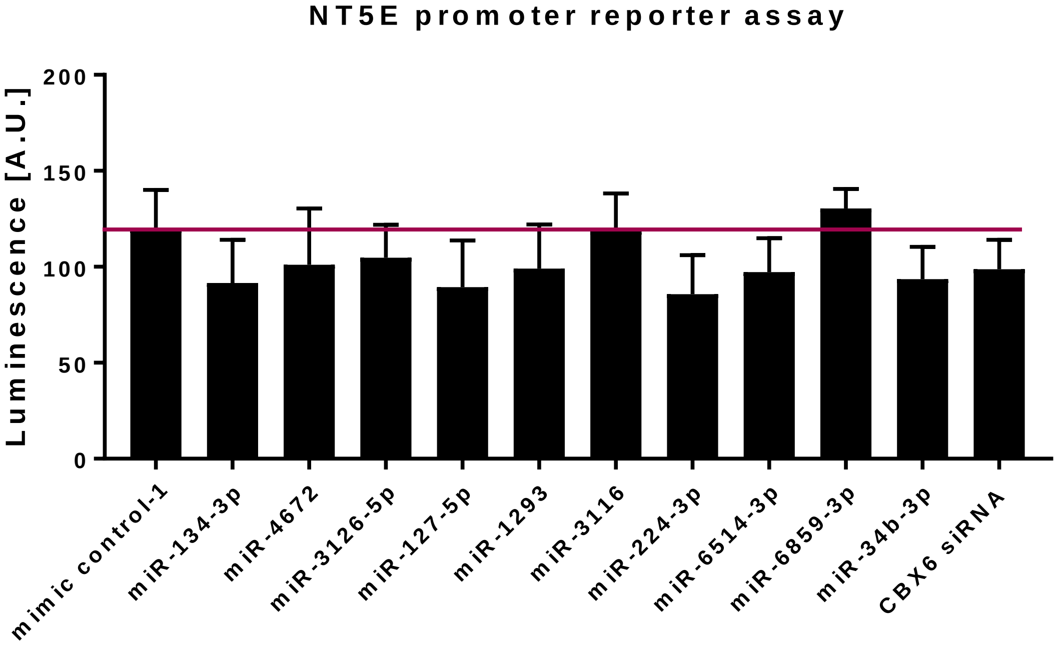


**Supplementary Figure 6: NT5E promoter reporter assay**. MDA-MB-231 cells were co-transfected with 50 nM miRNA or siRNA pools and 100 ng pLS-NT5E-prom vector (NT5E promoter fused to renilla luciferase gene). 72 h after treatment cells were lysed and luminescence was acquired to measure NT5E promoter activity. No significant effects were detected. Conditions were compared by One-way ANOVA comparing all conditions to mimic control-1.

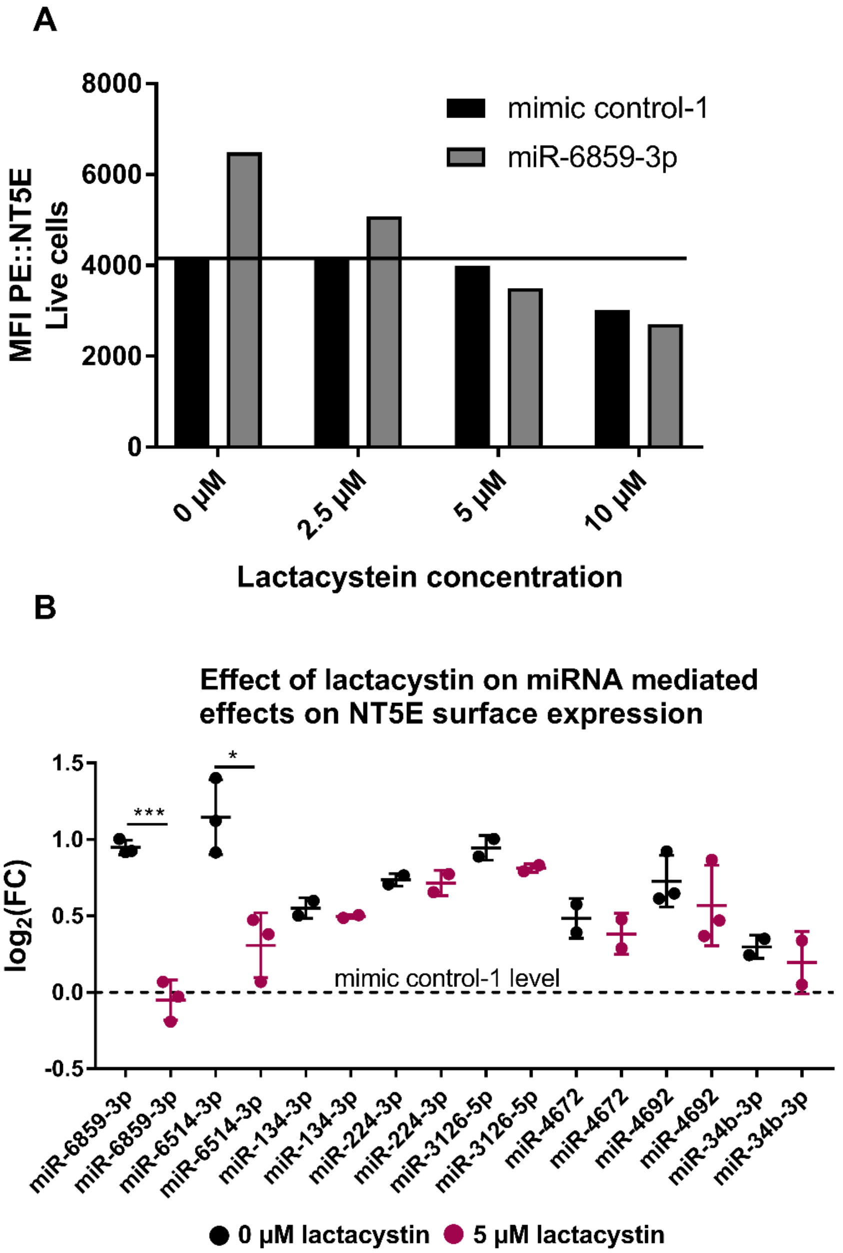


**Supplementary Figure 7: Effect of lactacystin on miRNA mediated effects on NT5E surface expression**. MDA-MB-231 cells were seeded in 12-well plates and after 24 h transfected with 50 nM miRNA. One day after transfection cells were treated with lactacystin. **(A)** The effect of lactacystin was titrated on MDA-MB-231 cells transfected with either mimic control-1 or miR-6859-3p miRNA. 5 µM lactacystin were selected for further experiments, since this concentration did not impact NT5E surface level for control miRNA **(B)**. Cells were either supplemented with 5 µM lactacystin (pink) or 0 µM lactacystin (black). Change in NT5E surface levels was measured by flow cytometry 72 h after transfection. Each dot represents an independent experiment. For each condition 2-3 replicates were performed. Samples were compared to respective mimic control-1 sample. Mean ± SD are shown. Significance was assessed by unpaired T-test. *: $p < 0.05$; **: $p < 0.01$; ***: $p < 0.001$; ****: $p < 0.0001$.

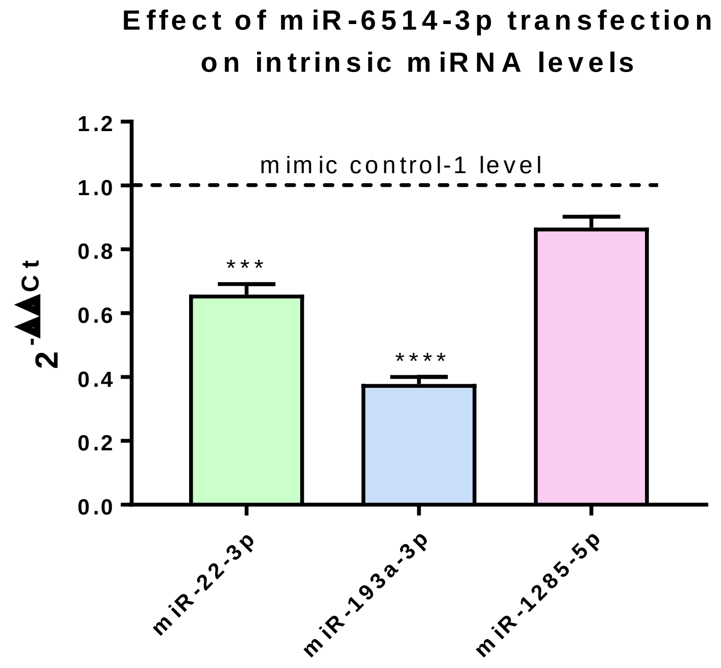


**Supplementary Figure 8: miR-6514-3p suppresses intrinsic level of miRNAs capable of inhibiting NT5E expression**. MDA-MB-231 cells were seeded in 12-well plates and after 24 h transfected with 50 nM miRNA. 48 h later cells were harvested and RNA was isolated using miRNeasy kit. miRNA levels were determined with miRNA-specific qPCR analysis using taqman-probes. RNU6B was used as house keeping control. Samples were normalized to mimic control-1 condition. Conditions were compared by One-way ANOVA comparing all conditions to mimic control-1. *: $p < 0.05$; **: $p < 0.01$; ***: $p < 0.001$; ****: $p < 0.0001$.

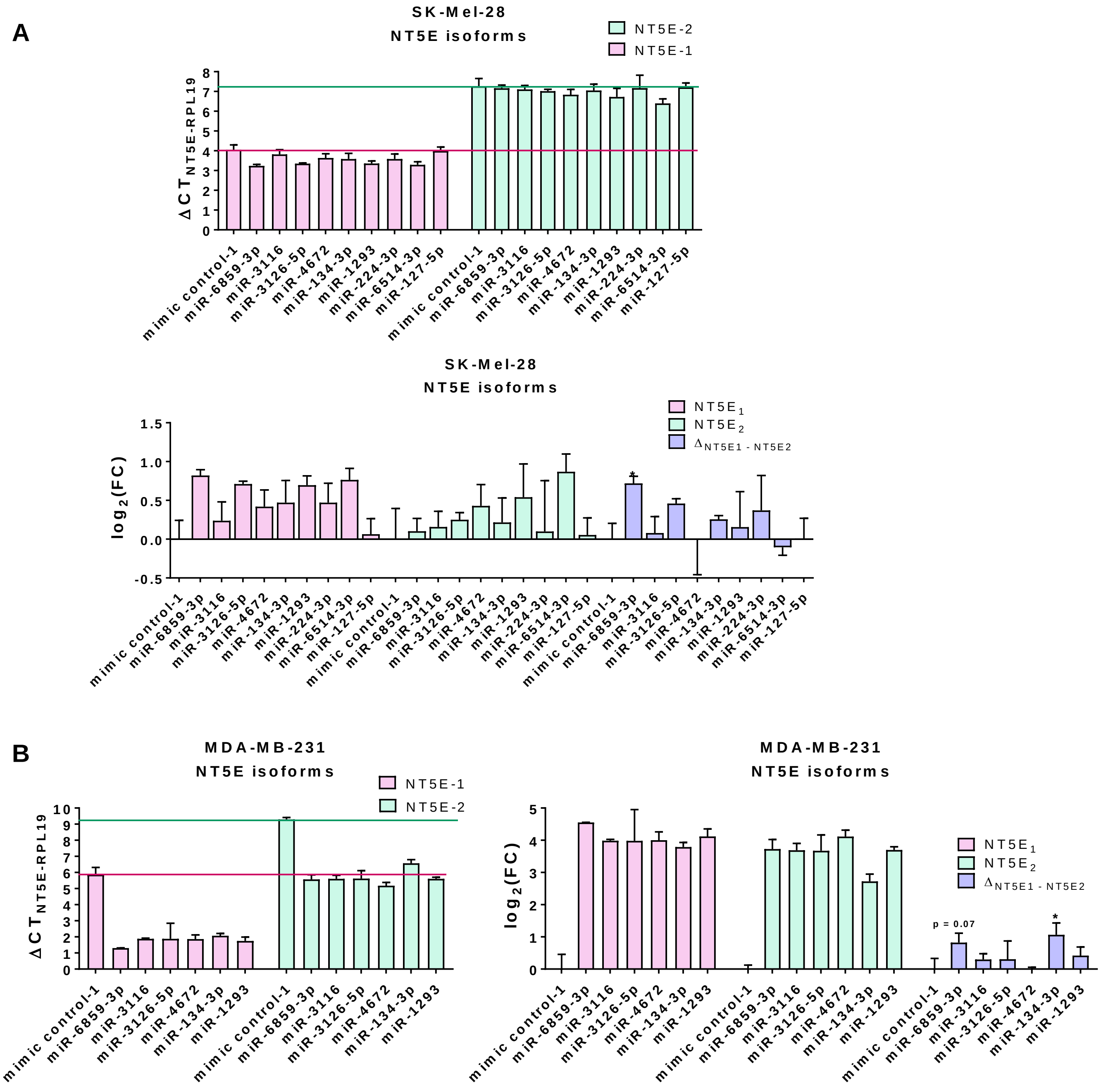


**Supplementary Figure 9: Effect of NT5E enhancing miRNAs on NT5E isoforms**. RNA extracts used for microarray analysis were reused to perform isoform-specific qPCR on SK-Mel-28 **(A)** and MDA-MB-231 cells **(B)**. RPL19 was used as housekeeping gene. ΔCTs were calculated for both NT5E isoforms: **NT5E-1** (normal, long variant) and **NT5E-2** (shorter variant). $Log_2$ Fold changes were calculated for both isoforms normalized to corresponding mimic control-1 samples. The differences of $log_2FC$ for NT5E-1 and NT5E-2 were calculated to estimate which miRNAs asymmetrically affect the two NT5E isoforms. Conditions were compared by One-way ANOVA comparing all conditions to mimic control-1. *: $p < 0.05$.

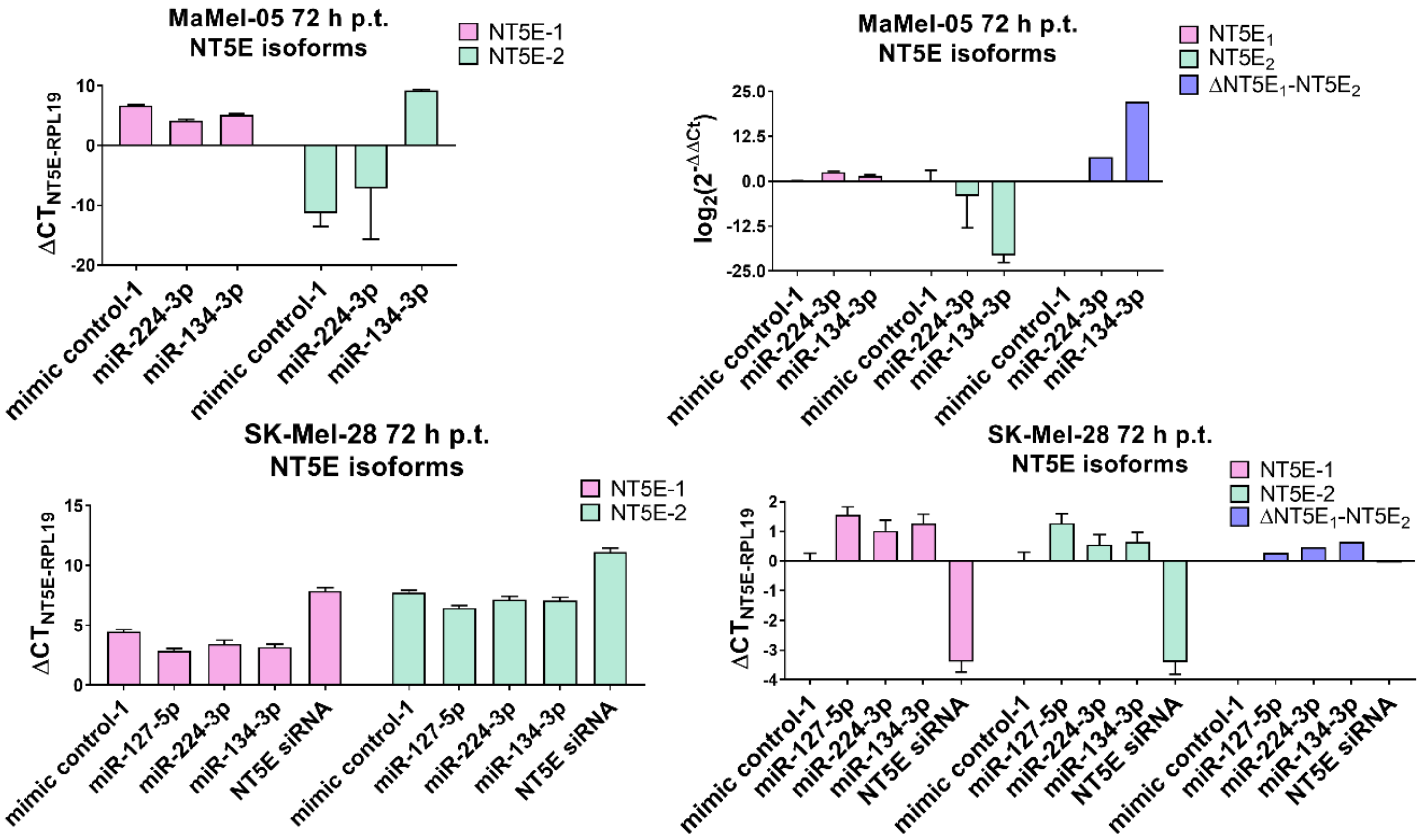


**Supplementary Figure 10: Effect of NT5E enhancing miRNAs on NT5E isoform expression**. SK-Mel-28 and MaMel-05 cells were transfected with 50 nM miRNA/siRNA and 72 h after transfection total RNA was isolated and used for isoform specific qPCR. RPL19 was used as housekeeping gene. $\Delta$CTs were calculated for both NT5E isoforms: **NT5E-1** (normal, long variant) and **NT5E-2** (shorter variant). Log$_2$ Fold changes were calculated for both isoforms normalized to corresponding mimic control-1 samples. The differences of log$_2$FC for NT5E-1 and NT5E-2 were calculated to estimate which miRNAs asymmetrically affect the two NT5E isoforms.

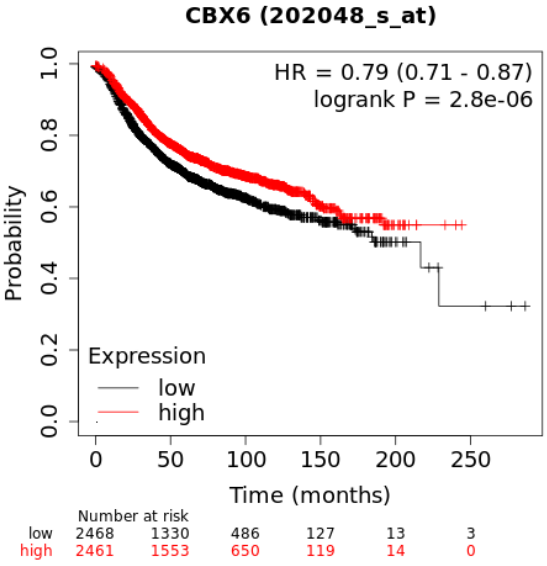
CBX6 (202048_s_at)
HR = 0.79 (0.71 - 0.87)
logrank P = 2.8e-06
Probability
Time (months)
Expression
low
high
Number at risk
low 2468 1330 486 127 13 3
high 2461 1553 650 119 14 0

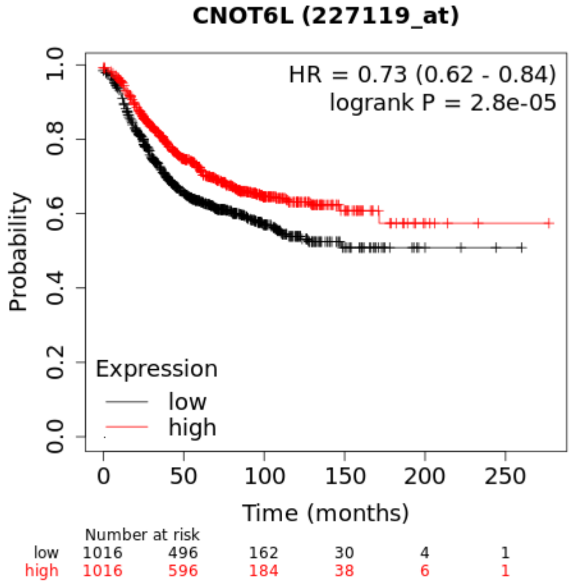
CNOT6L (227119_at)
HR = 0.73 (0.62 - 0.84)
logrank P = 2.8e-05
Probability
Time (months)
Expression
low
high
Number at risk
low 1016 496 162 30 4 1
high 1016 596 184 38 6 1

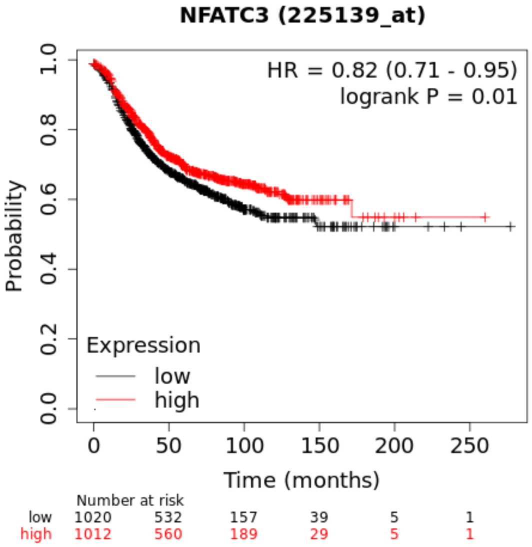
NFATC3 (225139_at)
HR = 0.82 (0.71 - 0.95)
logrank P = 0.01
Probability
Time (months)
Expression
low
high
Number at risk
low 1020 532 157 39 5 1
high 1012 560 189 29 5 1

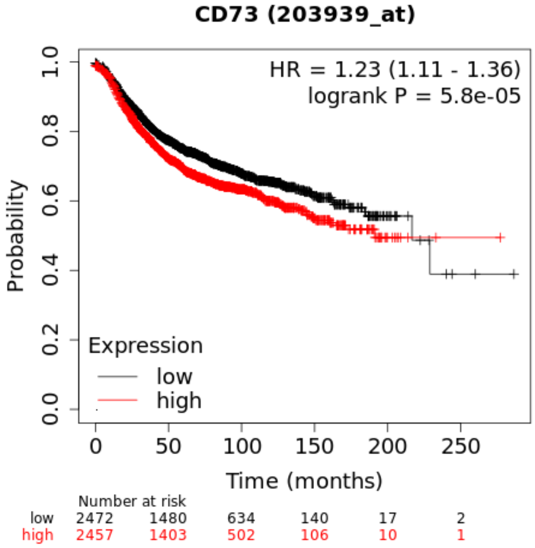
CD73 (203939_at)
HR = 1.23 (1.11 - 1.36)
logrank P = 5.8e-05
Probability
Time (months)
Expression
low
high
Number at risk
low 2472 1480 634 140 17 2
high 2457 1403 502 106 10 1

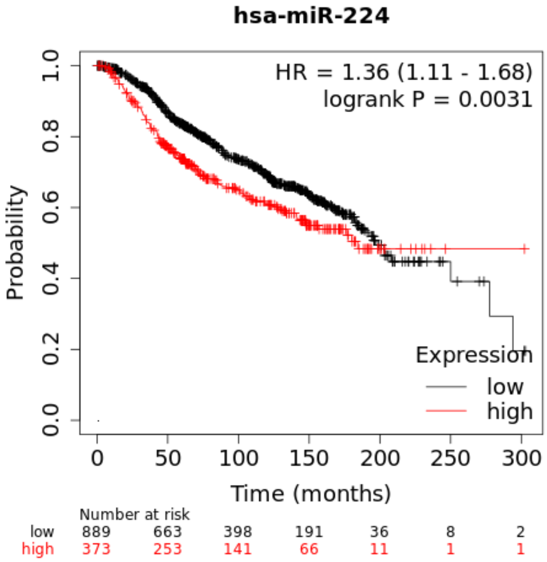
hsa-miR-224
HR = 1.36 (1.11 - 1.68)
logrank P = 0.0031
Probability
Time (months)
Expression
low
high
Number at risk
low 889 663 398 191 36 8 2
high 373 253 141 66 11 1 1

**Supplementary Figure 11: Kaplan-Meier analysis of CBX6 and CNOT6L in breast cancer patients.** High CBX6 expression is significantly associated with a better survival in human breast cancer patients (n = 4929). Also, high CNOT6L or NFATC3 mRNA levels are significantly associated with a better survival for breast cancer patients (n = 2032). In contrast, high NT5E/CD73 mRNA levels are significantly associated with worse prognosis for breast cancer patients. Furthermore, high miR-224 expression is linked to worse survival for breast cancer patients based on METABRIC data set (n = 1262). This indicates, that high levels of NT5E promoting miRNAs such as miR-224 or low expression of NT5E repressor such as CBX6/CNOT6L or NFACT3 are linked to progressive tumor disease reflected by shorter survival time. Plots were generated using Kaplan-Meier plotter tool [11].

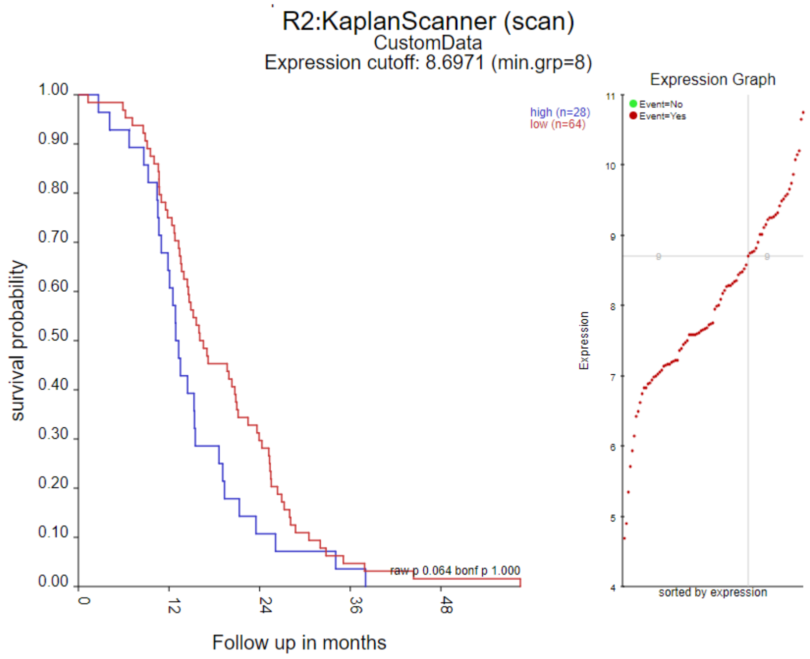


**Supplementary Figure 12: Kaplan-Meier analysis of miR-134 in melanoma patients.** High expression level of miR-134 levels shows a tendency to be associated with worse survival in human melanoma patients. Plot was generated using R2: Genomics Analysis and Visualization Platform (http://r2.amc.nl).

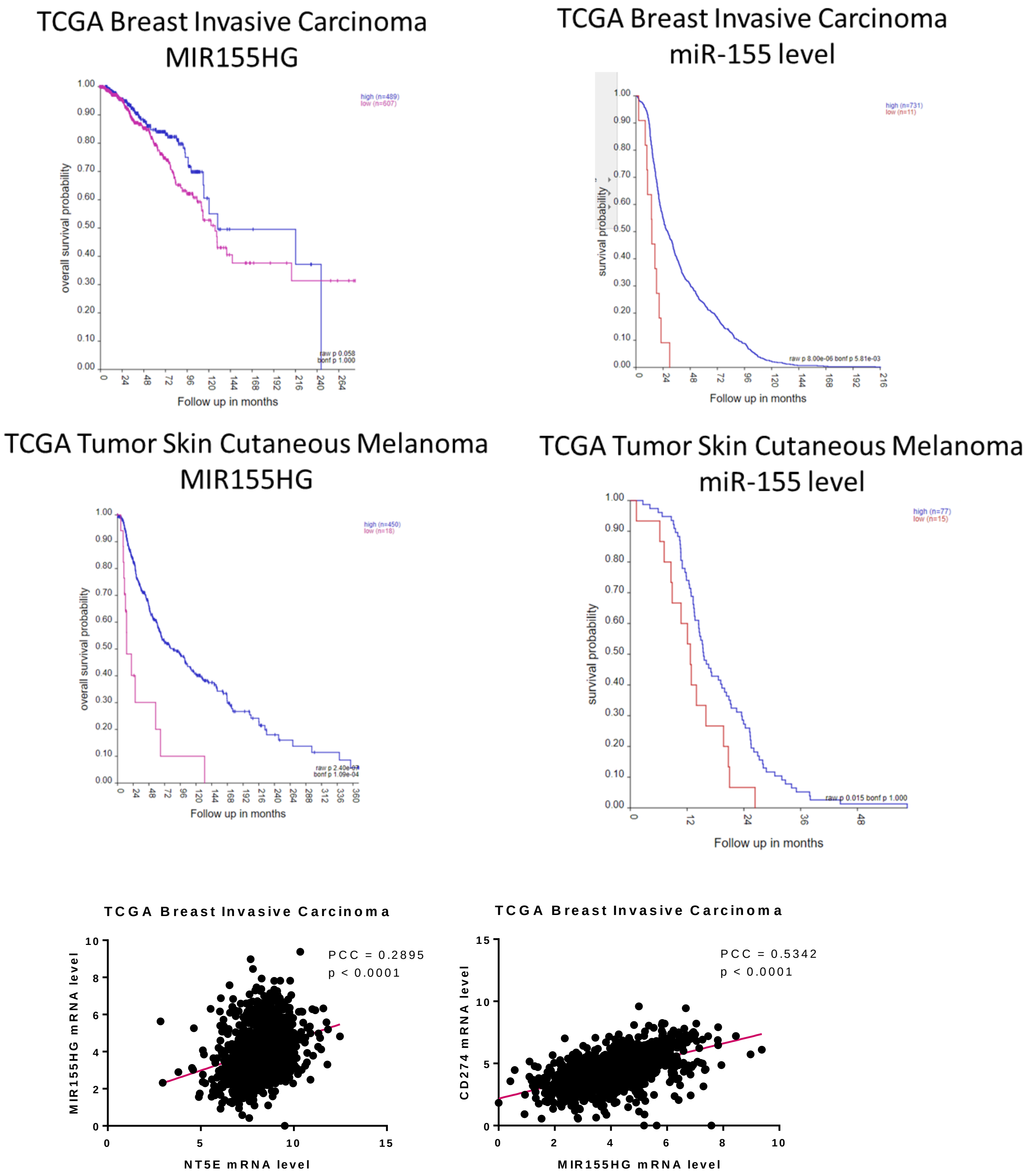
TCGA Breast Invasive Carcinoma
MIR155HG
overall survival probability
high (n=489)
low (n=607)
raw p 0.058
bonf p 1.000
Follow up in months
TCGA Breast Invasive Carcinoma
miR-155 level
survival probability
high (n=731)
low (n=11)
raw p 8.00e-06 bonf p 5.81e-03
Follow up in months
TCGA Tumor Skin Cutaneous Melanoma
MIR155HG
overall survival probability
high (n=450)
low (n=18)
Follow up in months
TCGA Tumor Skin Cutaneous Melanoma
miR-155 level
survival probability
high (n=77)
low (n=15)
raw p 0.015 bonf p 1.000
Follow up in months
TCGA Breast Invasive Carcinoma
MIR155HG mRNA level
PCC = 0.2895
p < 0.0001
NT5E mRNA level
TCGA Breast Invasive Carcinoma
CD274 mRNA level
PCC = 0.5342
p < 0.0001
MIR155HG mRNA level

**Supplementary Figure 13: Kaplan-Meier analysis of MIR155HG and miR-155 in breast cancer patients.** High expression level of MIR155HG gene is significantly associated with better survival in human melanoma patients. Also, in breast cancer the same tendency can be observed. Furthermore, high expression level of miRNA itself is significantly linked to better prognosis of both melanoma and breast cancer patients. Plots were generated using R2: Genomics Analysis and Visualization Platform (http://r2.amc.nl). Noteworthy, expression of NT5E mRNA and CD274 positively correlates with MIR155HG expression. This indicates a negative feedback loop, since miR-155-5p was proven to target and inhibit both CD274 and NT5E expression.

# References


1. Mu W, Zhang W. Bioinformatic Resources of microRNA Sequences, Gene Targets, and Genetic Variation. *Front Genet*. 2012;3:31. doi:10.3389/fgene.2012.00031
2. Betel D, Koppal A, Agius P, Sander C, Leslie C. Comprehensive modeling of microRNA targets predicts functional non-conserved and non-canonical sites. *Genome Biol*. 2010;11(8):R90. doi:10.1186/gb-2010-11-8-r90
3. Chen Y, Wang X. miRDB: an online database for prediction of functional microRNA targets. *Nucleic Acids Res*. Jan 8 2020;48(D1):D127-D131. doi:10.1093/nar/gkz757
4. Xiao F, Zuo Z, Cai G, Kang S, Gao X, Li T. miRecords: an integrated resource for microRNA-target interactions. *Nucleic Acids Res*. Jan 2009;37(Database issue):D105-10. doi:10.1093/nar/gkn851
5. Nam S, Kim B, Shin S, Lee S. miRGator: an integrated system for functional annotation of microRNAs. *Nucleic Acids Res*. Jan 2008;36(Database issue):D159-64. doi:10.1093/nar/gkm829
6. Hsu SD, Chu CH, Tsou AP, et al. miRNAMap 2.0: genomic maps of microRNAs in metazoan genomes. *Nucleic Acids Res*. Jan 2008;36(Database issue):D165-9. doi:10.1093/nar/gkm1012
7. Sulc M, Marin RM, Robins HS, Vanicek J. PACCMIT/PACCMIT-CDS: identifying microRNA targets in 3' UTRs and coding sequences. *Nucleic Acids Res*. Jul 1 2015;43(W1):W474-9. doi:10.1093/nar/gkv457
8. Krek A, Grun D, Poy MN, et al. Combinatorial microRNA target predictions. *Nat Genet*. May 2005;37(5):495-500. doi:10.1038/ng1536
9. Kertesz M, Iovino N, Unnerstall U, Gaul U, Segal E. The role of site accessibility in microRNA target recognition. *Nat Genet*. Oct 2007;39(10):1278-84. doi:10.1038/ng2135
10. Agarwal V, Bell GW, Nam JW, Bartel DP. Predicting effective microRNA target sites in mammalian mRNAs. *Elife*. Aug 12 2015;4doi:10.7554/eLife.05005
11. Gyorffy B. Survival analysis across the entire transcriptome identifies biomarkers with the highest prognostic power in breast cancer. *Comput Struct Biotechnol J*. 2021;19:4101-4109. doi:10.1016/j.csbj.2021.07.014
